# Chemical Constituents of the Root of *Jasminum giraldii*

**DOI:** 10.3390/molecules18044766

**Published:** 2013-04-22

**Authors:** Zhenggang Yue, Hui Qin, Yuhua Li, Yang Sun, Zhipeng Wang, Tiehong Yang, Li Liu, Minchang Wang, Feng Feng, Qibing Mei

**Affiliations:** 1 Department of Pharmacology, School of Pharmacy, the Fourth Military Medical University, Xi’an 710032, Shaanxi, China; 2 Collaborative Innovation Center for Chinese Medicine in Qingba Mountains, the Fourth Military Medical University, Xi’an 710032, Shaanxi, China; 3 Department of Natural Medicinal Chemistry, China Pharmaceutical University, Nanjing 210009, Jiangsu, China; 4 Xi’an Modern Chemistry Research Institute, Xi’an 710032, Shaanxi, China

**Keywords:** *Jasminum giraldii*, oleaceae, chemical constituents, cytotoxic activity

## Abstract

**abstract:** Two new compounds, ethylconiferin (**1**) and (−)-lariciresinol 4-(6'''-*O*-cinnamyl-*β*-D-glucopyranoside) (**2**), along with the three known compounds (+)-pinoresinol (**3**), (+)-cycloolivil (**4**), nobiletin (**5**), were isolated from the root of *Jasminum girialdii*. All these compounds were isolated for the first time from this source. Their structures were elucidated on the basis of extensive spectroscopic analysis and chemical methods. In addition, the *in vitro* cytotoxic activity of these compounds was evaluated. The results showed that none of the compounds had any significant inhibitory activities on the proliferation of HCT-116 and SW-620 cells.

## 1. Introduction

*Jasminum giraldii* Diels (Oleaceae), is an endemic plant which is found distributed at an altitude of 300–1,500 m, in valleys and shrubbery of the Qinba Mountains in China’s Shaanxi Province [1], Its dried roots, named as “quan pi” in Chinese, are used as a traditional local herb for the treatment of various diseases, such as fractures, traumatic injury and blood stasis, while some other species of this genus are employed to treat dysmenorrhea, metritis, leucorrhoea, galactophoritis, puerperal infection, irregular menstruation, hyperthermia, arthralgia, pain due to ischemia, dermatitis [2], fever, rheumatic pain [3], dysenteric diarrhea, trachelopanus, eczema [4], giddiness, edema [5] in Traditional Chinese Medicine.

**Figure 1:**
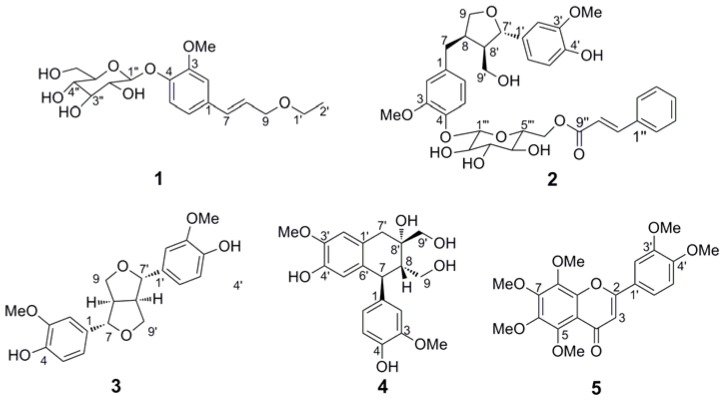
The structures of compounds **1**–**5**.

The chemistry and pharmacology of the ethanol extracts of the roots of *J. giraldii* have never been systematically investigated. As part of a program to acquire and assess potent chemicals in several traditional Chinese medicines for the preservation and treatment of colorectal cancer [6,7,8,9], an ethanolic extract of the root of *J. giraldii* has been investigated. We describe herein the isolation and structural elucidation of two new compounds **1**,**2** (Figure 1) along with three known compounds **3**–**5** [10,11,12]. Furthermore, the cytotoxic activity of these compounds against HCT-116 and SW-620 cells is reported for the first time. 

## 2. Results and Discussion

**Figure 2:**
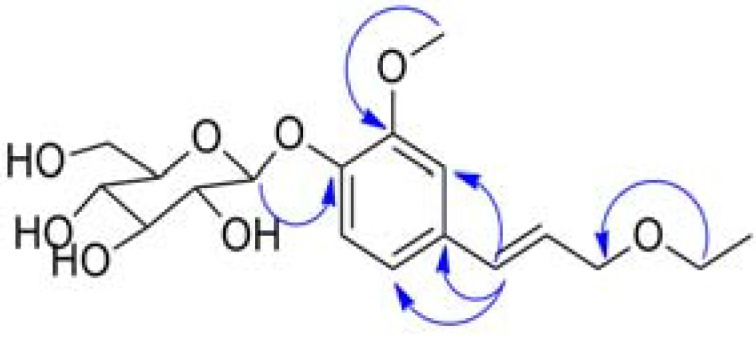
Key HMBC correlations in compound **1**.

Compound **1** was obtained as a white powder, whose molecular formula C_18_H_26_O_8_ was indicated by HR-TOF-MS at *m/z* 393.1525 [M+Na]^+^ (calcd for C_18_H_26_O_8_Na, 393.1525), implying six degrees of unsaturation. The max absorptions in the UV spectrum of **1** were observed at 290, 259, 213 nm (in CH_3_OH). The IR spectrum of **1** showed absorption bands of hydroxyl (3496, 3444, 3367 cm^−1^), aromatic ring (1510, 1454 cm^−1^), and olefinic (1649 cm^−1^) functionalities. The ^1^H-NMR spectra of **1** (Table 1) in CD_3_OD showed signals attributed to an aromatic ring ABX coupled system at *ä* 7.15(1H, d, *J* = 8.35 Hz, H-5), 7.11(1H, d, *J* = 1.90 Hz, H-2) and 6.99 (1H, dd, *J* = 1.90, 8.35 Hz, H-6), together with signals attributed to an aromatic methoxy group at *ä* 3.90 (3H, s), The presence of a *trans*-arylpropenoxy unit was characterized by signals at *ä* 6.61 (1H, d, *J* = 15.9 Hz, H-7), 6.26 (1H, dt, *J* = 6.15, 15.9 Hz, H-8) and 4.15 (2H, dd, *J* = 1.25, 6.15 Hz, H-9). Meanwhile, an ethoxyl unit was indicated by signals of 3.60 (2H, q, *J* = 7.05 Hz, H-1'), 1.25(3H, t, *J* = 7.05 Hz, H-2'), In addition, a doublet assignable to an anomeric proton at *δ* 4.93 (1H, d, *J* = 7.25 Hz, H-1''), together with partially overlapped signals attributed to oxymethylene and oxymethine protons at *δ* 3.90 (1H, dd, H-6a''), 3.72 (1H, dd, *J* = 7.45, 12.0 Hz, H-6b''), 3.52 (1H, m, H-2''), 3.51 (1H, m, H-3''), 3.50 (1H, m, H-5''), 3.48 (1H, m, H-4''), suggested that there was a glycosyl moiety with a *β* configuration in **1**. Enzymatic hydrolysis of **1** produced a sugar, which was identified as glucose by its ^1^H-NMR data and TLC comparison with an authentic sugar sample. The glucose isolated from the hydrolysate gave a positive optical rotation [α]^20^ *D*: +47.2, indicating that it was D-glucose [13]. The ^13^C-NMR and DEPT spectra of **1** showed carbon signals corresponding to the above units (Table 1). The HMBC correlation signals (Table 1 and Figure 2) of H-1'' (*δ*_H_ 4.93)/C-4 (*δ*_C_ 147.96) indicated that the glucosyl group was linked to C-4, H-7 (*δ*_H_ 6.61)/C-1 (*δ*_C_ 133.38), C-2 (*δ*_C_ 111.59), C-6 (*δ*_C_ 121.0) indicated that the *trans*-arylpropenoxy unit was connected to the aromatic ring via a C-7−C-1 bond, H-1' (*δ*_H_ 3.60)/C-9 (*δ*_C_ 72.38) indicated that the ethoxyl unit was linked to C-9. Therefore, compound **1**, whose ^1^H-NMR and ^13^C-NMR were similar to those of methylconiferin [14], was named ethylconiferin.

**Table 1:** .^1^H-NMR (500 MHz) and ^13^C-NMR (125 MHz) spectral data and HMBC Correlations of Compound **1** in CD_3_OD.

**NO.**	***δ*_C_**	***δ*_H_ (*J*, Hz)**	**HMBC**
**1**	133.38		
**2**	111.59	7.11 (1H, d, 1.9)	4, 6
**3**	151.07		
**4**	147.96		
**5**	118.08	7.15 (1H, d, 8.35)	1, 3
**6**	121.0	6.99 (1H, dd, 1.9, 8.35)	1, 2, 4, 5
**7**	133.55	6.61 (1H, d, 15.9)	1, 2, 6, 9
**8**	126.13	6.26 (1H, dt, 6.15, 15.85)	1, 9
**9**	72.38	4.15 (2H, dd, 1.25, 6.15)	1, 1', 8
**1'**	66.78	3.60 (2H, q, 7.05)	2', 9
**2'**	15.59	1.25 (3H, t, 7.05)	1'
**1''**	102.89	4.93 (1H, d, 7.25)	4
**2''**	75.05	3.52 (1H, m)	1'', 3''
**3''**	78.00	3.51 (1H, m)	2'', 4''
**4''**	71.49	3.48 (1H, m)	5''
**5''**	78.38	3.50 (1H, m)	
**6a'**	62.66	3.90 (1H, dd)	
**6b''**		3.72 (1H, dd, 7.45, 12)	
**3-OCH_3_**	56.87	3.90 (3H, s)	3

**Figure 3:**
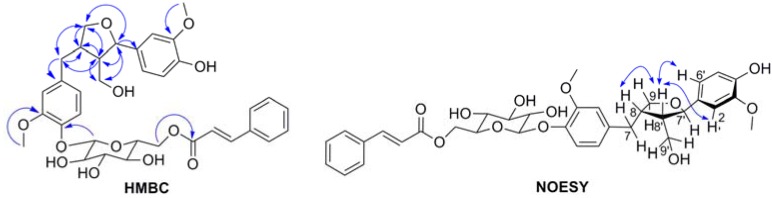
Key HMBC and NOESY correlations in compound **2**.

Compound **2** was isolated as a white powder, whose molecular formula C_35_H_40_O_12_ was determined by HR-TOF-MS at *m*/*z* 653.2589 [M+H]^+^ (calcd for C_35_H_41_O_12_, 652.2590), The UV absorption bands of **2** appeared at 278, 216 and 203 nm in CH_3_OH. The IR spectrum of **2** showed the presence of hydroxyl (3498, 3458, 3384 cm^−1^), conjugated-ester carbonyl (1714 cm^−1^), α,β-unsaturated olefinic (1645, 1635 cm^−1^) and aromatic ring (1512, 1456 cm^−1^) functionalities. The ^1^H-NMR data of **2** (Table 2) demonstrated signals attributable to two pairs of 1,3,4-trisubstituted benzene ring signals at *δ* 7.04 (1H, d, *J* = 8.25 Hz, H-5), 6.85 (1H, d, *J* = 1.75 Hz, H-2), 6.61 (1H, dd, *J* = 1.8, 8.25 Hz, H-6) and 6.90 (1H, d, *J* = 1.4 Hz, H-2'), 6.78 (1H, m H-5'), 6.77 (1H, m, H-6'); and a set of cinnamyl proton signals at *δ* 7.61 (2H ,m, H-2'', 6''), 7.43 (3H, m, H-3'', 4'', 5''), 7.68 (1H, d, *J* = 16 Hz, H-7''), 6.56 (1H, d, *J* = 16 Hz, H-8'') in the aromatic region. In addition, resonances assignable to two aromatic methoxyl proton signals at *δ* 3.86 (3H, s, 3'-OCH_3_), 3.85 (3H, s, 3-OCH_3_), and an anomeric proton signal at *δ* 4.88 (1H, d, H-1''') were observed. Furthermore, in the ^1^H-NMR spectrum, an *O*-bearing methine proton signals at *δ* 4.72 (1H, d, *J* = 6.65 Hz, H-7'), an aliphatic methylene proton signals at *δ* 2.85 (1H, dd, *J* = 4.7, 13.5 Hz, H-7a), 2.38 (1H, dd, 11.55, 13.3Hz, H-7b), two oxygenated methylene proton signals at *δ* 3.88(1H, dd, *J* = 6.5, 8.35 Hz, H-9a), 3.62 (1H, m, H-9b), 3.76 (1H, m, H-9a'), 3.60 (1H, m, H-9b'), and two aliphatic methine proton signals at *δ* 2.59 (1H, m, H-8), 2.31 (1H, m, H-8') were indicated. Besides the carbon resonances corresponding to the above units, the ^13^C-NMR spectrum (Table 2) and the DEPT indicated the presence of conjugated-ester carbonyl carbon signals at *δ* 168.37 (C-9''); a set of a *β*-glucose moiety carbon signals at *δ* 102.87 (C-1'''), 77.95 (C-3'''), 75.61 (C-5'''), 75.03 (C-2'''), 72.13 (C-4'''), 64.96 (C-6'''). The key HMBC correlations (Table 2 and Figure 3) from 3-OCH_3_ to C-3; from 3'-OCH_3_ to C-3'; from H-7 to C-1, C-2, C-6, C-8 and C-9; from H-9 to C-7, C-7', C-8 and C-8'; from H-7' to C-1', C-2', C-6', C-8', C-9 and C-9'; from H-8' to C-1', C-7, C-7', C-8, C-9 and C-9'; and from H-9' to C-7, C-7', C-8 and C-8'; suggested that the aglycone should be lariciresinol [15]. Moreover, the HMBC correlations of H-1'''/C-4 confirmed that anomeric protons of *β*-glucose moiety was coupled with C-4. Meanwhile, the more deshielded proton of H-6''' (*δ*_H_ 4.55 and 4.33) exhibited a HMBC correlation with C-9'', which confirmed that the cinnamyl moiety was linked to C-6'''. In the NOESY spectrum of **2** (Figure 3), H-8' showed NOE correlations with H-2', H-6' and H-8, this experiment confirmed that H-8 and H-8' are in *cis*-configuration, meanwhile, H-7' and H-8' are in *trans*-configuration. Comparison of the NMR data of (−)-lariciresinol 4'-(6''-*O*-feruloyl-*β*-D-glucopyranoside) [16] and compound **2** indicated they had very similar aglycone and sugar moieties, the only difference being the presence of a cinnamyl moiety instead of a feruloyl group. Compound **2** was hydrolysed by dilute acid, and the hydrolysate was identified to be D-glucose by the same way as described in the case of **1**, Therefore the structure of **2** was determined to be (−)-lariciresinol 4-(6'''-*O*-cinnamyl-*β*-D-glucopyranoside).

**Table 2:** .^1^H-NMR (500 MHz) and ^13^C-NMR (125 MHz) spectral data and HMBC correlations of compound **2** in CD_3_OD.

**No.**	***δ*_C_**	***δ*_H_ (*J*, Hz)**	**HMBC**
**1**	137.19		
**2**	114.46	6.85 (1H, d, 1.75)	1, 3, 4, 6, 7
**3**	150.95		
**4**	146.21		
**5**	118.42	7.04 (1H, d, 8.25)	1, 2, 3, 4
**6**	122.34	6.61 (1H, dd, 1.8, 8.25)	2, 3, 4, 5, 7
**7a**	33.85	2.85 (1H, dd, 4.7, 13.5)	1, 2, 6, 8, 9
**7b**		2.38 (1H, dd, 11.55, 13.3)	1, 2, 6, 8
**8**	43.66	2.59 (1H, m)	
**9a**	73.53	3.88 (1H, dd, 6.5, 8.35)	7, 7', 8'
**9b**		3.62 (1H, m)	7, 7', 8, 8'
**1'**	135.87		
**2'**	110.78	6.90 (1H, d, 1.4)	1', 3', 4', 6', 7'
**3'**	149.14		
**4'**	147.2		
**5'**	116.15	6.78 (1H, m)	1', 3', 4'
**6'**	119.93	6.77 (1H, m)	2', 8'
**7'**	84.11	4.72 (1H, d, 6.65)	1', 2', 6', 8', 9, 9'
**8'**	54.15	2.31 (1H, m)	1', 7, 7', 8, 9, 9'
**9a'**	60.59	3.76 (1H, m)	7, 7', 8, 8'
**9b'**		3.60 (1H, m)	7, 7', 8, 8'
**1''**	135.90		
**2'',6''**	129.47	7.61 (2H, m)	4'', 7''
**3'',5''**	130.28	7.43 (2H, m)	1''
**4''**	131.75	7.43 (1H, m)	1'', 3'', 5''
**7''**	146.59	7.68 (1H, d, 16)	1'', 2'', 6'', 8'', 9''
**8''**	118.96	6.56 (1H, d, 16)	1'', 9''
**9''**	168.37		
**1'''**	102.87	4.88 (1H, d)	4
**2'''**	75.03	3.53 (1H, m)	1''', 4'''
**3'''**	77.95	3.52 (1H, m)	2''', 4'''
**4'''**	72.13	3.43 (1H, t)	3''', 5''', 6'''
**5'''**	75.61	3.72 (1H, m)	1''', 4''', 6'''
**6a'''**	64.96	4.55 (1H, dd, 2.2, 11.75)	5''', 9''
**6b'''**		4.43 (1H, dd, 7.4, 11.65)	5''', 9''
**3-OCH_3_**	56.85	3.85	3
**3’-OCH_3_**	56.55	3.86	3'

The known compounds **3**–**5** were identified by comparison of their spectroscopic data with literature values. HCT-116 and SW-620 cells were treated with three different concentrations of compounds for 24 h. The antiproliferative effects of the five compounds on HCT-116 and SW-620 cells are shown in Table 3. None of the tested compounds had any significant effects on proliferation of HCT-116 cells and SW-620 cells at the tested concentrations of 1–10 μM.

**Table 3:** Activity of compounds **1**–**5** on proliferation of the HCT-116 and SW-620 cells.

	HCT-116 cells			SW-620 cells		
Sample	10^−4^ mol/L	10^−5^ mol/L	10^−6^ mol/L	10^−4^ mol/L	10^−5^ mol/L	10^−6^ mol/L
	Inhibitory rate (%)	Inhibitory rate (%)	Inhibitory rate (%)	Inhibitory rate (%)	Inhibitory rate (%)	Inhibitory rate (%)
1	5.383 ± 2.187 **	16.150 ± 0.504 **	13.329 ± 0.367 **	6.967 ± 0.262 **	5.026 ± 0.713 **	1.218 ± 1.016 **
2	13.049 ± 1.607 **	14.685 ± 1.615 **	11.671 ± 0.941 **	12.260 ± 0.443 **	3.141 ± 1.101 **	2.894 ± 0.461 **
3	12.086 ± 1.404 **	13.157 ± 0.748 **	5.943 ± 0.931 **	24.119 ± 1.128 **	8.605 ± 0.745 **	5.863 ± 0.287 **
4	14.750 ± 2.171 **	6.288 ± 1.735 **	3.725 ± 0.914 **	24.481 ± 0.184 **	2.894 ± 0.960 **	6.035 ± 0.605 **
5	19.079 ± 0.440 **	13.868 ± 0.373 **	2.972 ± 0.734 **	26.499 ± 0.453 **	3.769 ± 0.799 **	2.646 ± 1.577 **
Control	0	0	0	0	0	0

The data are expressed as mean ± SD of three independent experiments. (** *p* < 0.01 *vs*. control).

## 3. Experimental

### 3.1. General

UV spectra were taken with a HALO DB-20R UV-VIS spectrophotometer. Optical rotations were recorded on a PerkinElmer Model 343 polarimeter. The IR spectra were recorded on a Shimadzu FTIR-8400S instrument. ESI-MS was performed on s Waters Quattro Premier instrument. The HR-ESIMS spectra was taken on an Agilent Technologies 6550 Q-TOF. 1D and 2D NMR spectra were recorded on a Bruker-AVANCE500 instrument with TMS as an internal standard. The analytical HPLC was performed on a Waters 2695 Separations Module coupled with a 2996 Photodiode Array Detector and a ODS-3 column (4.6 × 250 mm, 5 ìm particles, Inertsill, Tokyo, Japan). Semipreparative HPLC was performed on a system comprising a Shimadzu LC-6AD pump equipped with a SPD-20A UV detector and a Ultimate XB-C18 (10 × 250 mm, 5 μm particles) or Allsphere ODS-2 (10 × 250 mm, 5 μm particles). Sephadex LH-20 was purchased from GE Healthcare Bio-Sciences AB (Uppsala, Sweden). MCI GEL was from Mitsubishi Chemical Corporation (Tokyo, Japan). C-18 (40–75 μm) silicagel was purchased from SiliCycle Corporation (Quebec, Canada). D101 was from Sunresin New Materials Co. Ltd. (Xi’an, China). Silica gel was purchased from Qingdao Haiyang Chemical Group Corporation (Qingdao, China).

### 3.2. Plant Materials

The root of *Jasminum giraldii* Diels. was collected on October in 2009 in Shanxi, China, and authenticated by Prof. Yaowu Guo. A voucher specimen has been deposited in the Department of Collaborative Innovation Center for Chinese Medicine in Qingba Mountains, School of Pharmacy the Fourth Military Medical University.

### 3.3. Extraction and Isolation

The root of *J. giraldii* (20 kg) was powdered and extracted with EtOH (100 L, 3 h × 4) under reflux. Evaporation of the solvent under reduced pressure gave the EtOH extract. The combined extract was applied to D101 and eluted with EtOH-H_2_O to obtain the 95% EtOH eluate (15 L). The eluate was evaporated under vacuum and freeze dried to yield a powder (895 g), which was suspended in H_2_O and partitioned successively with petroleum ether, EtOAc and *n*-BuOH. The EtOAc fraction was subjected to silica-gel column chromatography eluting with CHCl_3_-MeOH (100:0 to 1:1) to obtain 33 fractions (Fr.1–Fr.33). Fr.28 was further separated by flash chromatography over MCI, eluting with a gradient of MeOH (0–100%) in H_2_O to obtain seven fractions (Fr.28-1–Fr.28-7). Repeated chromatography of Fr.28-1 with silica gel (MeOH/CHCl_3_, 30:1–5:1), Sephadex LH-20 (CHCl_3_/MeOH, 1:1), prep-TLC and prep-HPLC gave compounds **1** (8 mg), **2** (26 mg), **4** (28 mg), Fr.21 was subjected to silica gel (200–300 mesh) column chromatography and eluted with CHCl_3_/MeOH (100:0–50:1) to obtain five fractions (Fr.21-1–Fr.21-5). Fr.21-5 was further separated repeated with silica gel column (petroleum ether/acetone, 8:1–5:1), Sephadex LH-20 (CHCl_3_/MeOH, 1:1), prep-TLC and prep-HPLC to afford compounds **3 **(120 mg) and **5** (5 mg).

### 3.4. Spectral Data

*Ethylconiferin* (**1**): white powder; C_18_H_26_O_8_; ${\left[\alpha \right]}_{D}^{20}$: −19.76 (c 0.1, CH_3_OH); UV_max_ (MeOH) *λ*_max_ 290, 259 and 213 nm; IR (KBr) ν_max_ 3496, 3444, 3367, 2979, 2939, 2867, 1649, 1510, 1454, 1128, 1089, 1047, 1027 cm^−1^; HR-TOF-MS *m/z* 393.1525 [M+Na]^+^ (calcd for C_18_H_26_NaO_8_; 393.1525); ^1^H-NMR (500 MHz, CD_3_OD) and ^13^C-NMR (125 MHz, CD_3_OD) data, see Table 1.

*(−)-Lariciresinol 4-(6'''-O-cinnamyl-β-D-glucopyranoside)* (**2**): white powder; C_35_H_40_O_12_; ${\left[\alpha \right]}_{D}^{20}$: −10.91 (c 0.1, MeOH); UV_max_ (MeOH) *λ*_max_ 278, 216 and 203 nm; IR(KBr) ν_max_ 3498, 3458, 3384, 2925, 2854, 1714, 1645, 1635, 1512, 1456, 1269, 1226, 1161, 1124, 1105, 1072, 1035 cm^−1^; HR-TOF-MS *m/z* 653.2590 [M+H]^+^ (calcd for C_35_H_41_O_8_; 653.2590); ^1^H-NMR (500 MHz, CD_3_OD) and ^13^C-NMR (125 MHz, CD_3_OD) data, see Table 2.

*(+)-Pinoresinol* **(3)**: yellow powder; C_20_H_22_O_6_; ${\left[\alpha \right]}_{D}^{20}$: +46.0 (c 0.1, MeOH); UV_max_ (MeOH) *λ*_max_ 280, 231 and 207 nm; IR (KBr) ν_max_ 3504, 3438, 3379, 2958, 2939, 2860, 1604, 1514, 1460, 1438, 1271, 1029 cm^−1^; ESI-MS *m/z* 357 [M−H]^−^; ^1^H-NMR (500 MHz, Me_2_CO-*d_6_*) *δ* 7.47 (2H, s, 4-OH and 4'-OH), 6.99 (2H, d, *J* = 1.7 Hz, H-2, 2'), 6.84 (2H, dd, *J* = 1.7, 8.1 Hz, H-6, 6'), 6.79 (2H, d, *J* = 8.1 Hz, H-5, 5'),4.67 (2H, d, *J* = 4.15 Hz, H-7, 7'), 4.20 (2H, dd, *J* = 6.95, 8.95 Hz, H-9a, 9'a), 3.84(6H, s, 3-OCH_3_ and 3'-OCH_3_), 3.81 (2H, dd, *J* = 3.7, 9.1 Hz, H-9b, 9'b), 3.08 (2H, m, H-8, 8'); ^13^C-NMR (125 MHz, Me_2_CO-*d_6_*) *δ* 148.40 (C-3, 3'), 146.94 (C-4, 4'), 134.27 (C-1, 1'), 119.69 (C-6, 6'), 115.62 (C-5, 5'), 110.67 (C-2, 2'), 86.72 (C-7, 7'), 72.29 (C-9, 9'), 56.33 (3-OCH_3_ and 3'-OCH_3_), 55.32 (C-8, 8').

*(+)-Cycloolivil* (**4**): yellow powder; C_20_H_24_O_7_; ${\left[\alpha \right]}_{D}^{20}$: +43.2 (c 0.1, MeOH); UV_max_ (MeOH) *λ*_max_ 283, 230 and 204 nm; IR (KBr) ν_max_ 3517, 3444, 3313, 3201, 2939, 2900, 2835, 1699, 1598, 1517, 1465, 1446, 1433, 1373, 1269, 1236, 1215, 1149, 1124, 1022, 881, 858, 838, 811 cm^−1^; ESI-MS *m/z* 375 [M−H]^−^; ^1^H-NMR (500 MHz, CD_3_OD) *δ* 6.76 (1H, d, *J* = 8.0 Hz, H-5), 6.76 (1H, d, *J* = 1.6 Hz, H-2), 6.67 (1H, dd, *J* = 1.7, 7.95 Hz, H-6), 6.63 (1H, s, H-5'), 6.18 (1H, s, H-2'), 4.03 (1H, d, *J* = 11.65 Hz, H-7), 3.80 (3H, s, OCH_3_), 3.77 (2H, m, H-9a, 9'a), 3.74 (3H, OCH_3_), 3.59 (2H, m, H-9b, 9'b), 3.23 (1H, d, *J* = 16.65 Hz, H-7'a), 2.62 (1H, d, *J* = 16.65 Hz, H-7'b), 2.04 (1H, m, H-8); ^13^C-NMR (125 MHz, CD_3_OD) *δ* 149.27 (C-3), 147.64 (C-3'), 146.25 (C-4), 145.44 (C-4'), 138.62 (C-1'), 133.69 (C-1), 126.57 (C-6'), 123.71 (C-6), 117.48 (C-5), 116.15 (C-5'), 114.06 (C-2), 113.09 (C-2'), 75.10 (C-8'), 69.55 (C-9), 60.98 (C-9'), 56.52 (OCH_3_), 56.49 (OCH_3_), 47.69 (C-8), 45.03 (C-7), 40.06 (C-7').

*Nobiletin* (**5**): white powder; C_9_H_12_O_2_; ${\left[\alpha \right]}_{D}^{20}$: +3.69 (c 0.1, MeOH); UV_max_ (MeOH) *λ*_max_ 328, 269, 249 and 208 nm. IR (KBr) ν_max_ 2947, 2854, 1629, 1598, 1514, 1461, 1377, 1278, 1149, 1107, 1078, 1012, 973, 838 cm^−1^; ESI-MS *m/z* 403 [M+H]^+^; ^1^H-NMR (500 MHz, Me_2_CO-*d_6_*) *δ* 7.66 (1H, dd, *J* = 2.15, 8.5 Hz, H-6'), 7.59 (1H, d, *J* = 2.05 Hz, H-2'), 7.15 (1H, d, *J* = 8.5 Hz, H-5'), 6.62 (1H, s, H-3), 4.07 (3H, s, OCH_3_), 4.04 (3H, s, OCH_3_), 3.96 (3H, s, OCH_3_), 3.92 (3H,s, OCH_3_), 3.89 (3H, s, OCH_3_), 3.85 (3H, s, OCH_3_); ^13^C-NMR (125 MHz, Me_2_CO-*d_6_*) *δ* 176.74 (C-4), 161.67 (C-2), 153.38 (C-4'), 152.4 (C-7), 150.72 (C-3'), 149.22 (C-9), 148.64 (C-8), 145.15 (C-5), 139.24 (C-6), 124.85 (C-1'), 120.39 (C-6'), 115.91 (C-10), 112.69 (C-5'), 110.01 (C-2'), 107.29 (C-3), 62.44, 62.37, 62.04, 61.98, 56.34, 56.31(5-OCH_3_ 6-OCH_3_, 7-OCH_3_, 8-OCH_3_, 3'-OCH_3_ and 4'-OCH_3_,).

### 3.5. Enzymatic Hydrolysis of **1**

A solution of compound **1** in H_2_O (3 mL) was hydrolysed with β-glucosidase (10 mg, Almonds Lot 1264252, Sigma-Aldrich, St. Louis, MO, USA) at 37 °C for 24 or 36 h. The reaction mixture was extracted with EtOAc for three times (3 × 3 mL) to yield the EtOAc exrract and H_2_O phase after removing the solvents. The aqueous phase of the hydrolysate of compound 1 was subjected to CC over silica gel eluted with MeCN/H_2_O (8:1) to yield glucose with positive optical rotations, and the value of ${\left[\alpha \right]}_{D}^{20}$: was +47.2 (c 0.16, H_2_O). The solvent system MeCN/H_2_O (6:1) was used for the TLC analysis of glucose and authentic sugar samples.

### 3.6. Acid Hydrolysis of **2**

A solution of compound **2** in 0.5 M H_2_SO_4_ (2.0 mL) was heated under reflux for 3 h. After cooling, the reaction mixture was diluted with H_2_O, neutralized with BaCO_3_, then filtered. The solution was extracted with EtOAc for three times (3 × 3 mL) to obtained the EtOAc extract and H_2_O layer after removing the solvents. The aqueous layer of the hydrolysate of compound **2** was evaporated and to yield glucose with positive optical rotations, and the value of ${\left[\alpha \right]}_{D}^{20}$: was +42.5 (*c* 0.11, H_2_O). The solvent system MeCN/H_2_O (6:1) was used for the TLC analysis of glucose and authentic sugar samples.

### 3.7. Bioassay for Cytotoxic Activity

The human colon cell line HCT-116 and SW620 was obtained from the Shanghai Institute of Cell Biology. The cells were maintained in DMEM medium supplemented with 10% FBS, penicillin (100 U/mL) and streptomycin (100 mg/mL) at 37 °C in a humidified atmosphere with 5% CO_2_. Cell proliferation of HCT-116 cells was assessed by conducting colorimetric MTT cell proliferation assay. Briefly, a limited number of growing cells were seeded into 96-well cell culture plate and were maintained for 24 h so that they became attached to the bottom of the well. The medium was aspirated and new medium (200 µL) including three groups of concentrations (10^−4^, 10^−5^, 10^−6^ M) of compounds were added to parallel wells. The cells were incubated at 37 °C in a humidified atmosphere for 24 h. Twenty µL of MTT solution was added and the incubation continued for 4 h. The pure formazan product was then solubilized by 150 µL DMSO. The plates were read at 570 nm using a microtiter plate reader.

## 4. Conclusions

Compounds **1**, **2** were new glycosides and together with compounds **3**–**5** were isolated from the *Jasminum* genus for the first time. Compounds **1**–**5** were assayed for their cytotoxic activity, and the data proved that none of the compounds had any significant inhibitory activities on proliferation of HCT-116 cells and SW-620 cells at the concentrations of 1–10 μM.

## Supplementary Materials

Supplementary materials can be accessed at: http://www.mdpi.com/1420-3049/18/4/4766/s1.
