# Supplementary material for: Chemical Constituents of the Root of Jasminum giraldii
Source: Molecules. 2013 Apr 22;18(4):4766–75. doi: 10.3390/molecules18044766 (PMC6270083; doi:10.3390/molecules18044766)

# Supplementary Materials

## List of Content


| No. | Content | page |
|---|---|---|
| 1 | **Figure S1.** The structures of Compounds **1–5** | S3 |
| 2 | **Figure S2.** Main HMBC Correlations of Compounds **1** and **2** | S3 |
| 3 | **Figure S3.** Main NOESY Correlations of Compound **2** | S3 |
| 4 | **Figure S4.** The UV Spectrum of Compound **1** in $CH_3OH$ | S4 |
| 5 | **Figure S5.** The IR Spectrum of Compound **1** | S5 |
| 6 | **Figure S6.** The HR-QTF-MS Spectrum of Compound **1** | S6 |
| 7 | **Figure S7.** The $^1$H--NMR Spectrum of Compound **1** in $CD_3OD$ | S7 |
| 8 | **Figure S8.** The $^{13}$C-NMR Spectrum of Compound **1** in $CD_3OD$ | S8 |
| 9 | **Figure S9.** The DEPT Spectrum of Compound **1** in $CD_3OD$ | S9 |
| 10 | **Figure S10.** The HMQC Spectrum of Compound **1** in $CD_3OD$ | S10 |
| 11 | **Figure S11.** The HMBC Spectrum of Compound **1** in $CD_3OD$ | S11 |
| 12 | **Figure S12.** The UV Spectrum of Compound **2** in $CH_3OH$ | S12 |
| 13 | **Figure S13.** The IR Spectrum of Compound **2** | S13 |
| 14 | **Figure S14.** The HR-QTF-MS Spectrum of Compound **2** | S14 |
| 15 | **Figure S15.** The $^1$H-NMR Spectrum of Compound **2** in $CD_3OD$ | S15 |
| 16 | **Figure S16.** The $^{13}$C-NMR Spectrum of Compound **2** in $CD_3OD$ | S16 |
| 17 | **Figure S17.** The DEPT Spectrum of Compound **2** in $CD_3OD$ | S17 |
| 18 | **Figure S18.** The HMQC Spectrum of Compound **2** in $CD_3OD$ | S18 |
| 19 | **Figure S19.** The HMBC Spectrum of Compound **2** in $CD_3OD$ | S19 |
| 20 | **Figure S20.** The NOESY Spectrum of Compound **2** in $CD_3OD$ | S20 |
| 21 | **Figure S21.** The NOESY Spectrum of Compound **2** in $CD_3OD$ | S21 |
| 22 | **Figure S22.** The UV Spectrum of Compound **3** in $CH_3OH$ | S22 |
| 23 | **Figure S23.** The IR Spectrum of Compound **3** | S23 |
| 24 | **Figure S24.** The MS Spectrum of Compound **3** | S24 |
| 25 | **Figure S25.** The $^1$H-NMR Spectrum of Compound **3** in $Me_2CO–d_6$ | S25 |
| 26 | **Figure S26.** The $^{13}$C-NMR Spectrum of Compound **3** in $Me_2CO–d_6$ | S26 |
| 27 | **Figure S27.** The UV Spectrum of Compound **4** in $CH_3OH$ | S27 |
| 28 | **Figure S28.** The IR Spectrum of Compound **4** | S28 |
| 29 | **Figure S29.** The MS Spectrum of Compound **4** | S29 |
| 30 | **Figure S30.** The $^1$H-NMR Spectrum of Compound **4** in $CD_3OD$ | S30 |
| 31 | **Figure S31.** The $^{13}$C-NMR Spectrum of Compound **4** in $CD_3OD$ | S31 |
| 32 | **Figure S32.** The DEPT Spectrum of Compound **4** in $CD_3OD$ | S32 |
| 33 | **Figure S33.** The UV Spectrum of Compound **5** in $CH_3OH$ | S33 |
| 34 | **Figure S34.** The IR Spectrum of Compound **5** | S34 |
| 35 | **Figure S35.** The ESIMS Spectrum of Compound **5** | S35 |
| 36 | **Figure S36.** The $^1$H-NMR Spectrum of Compound **5** in $Me_2CO–d_6$ | S36 |
| 37 | **Figure S37.** The $^{13}$C-NMR Spectrum of Compound **5** in $Me_2CO–d_6$ | S37 |
| 38 | **Figure S38.** The DEPT Spectrum of Compound **5** in $Me_2CO–d_6$ | S38 |

**Figure S1.** The structures of compounds **1**–**5**.

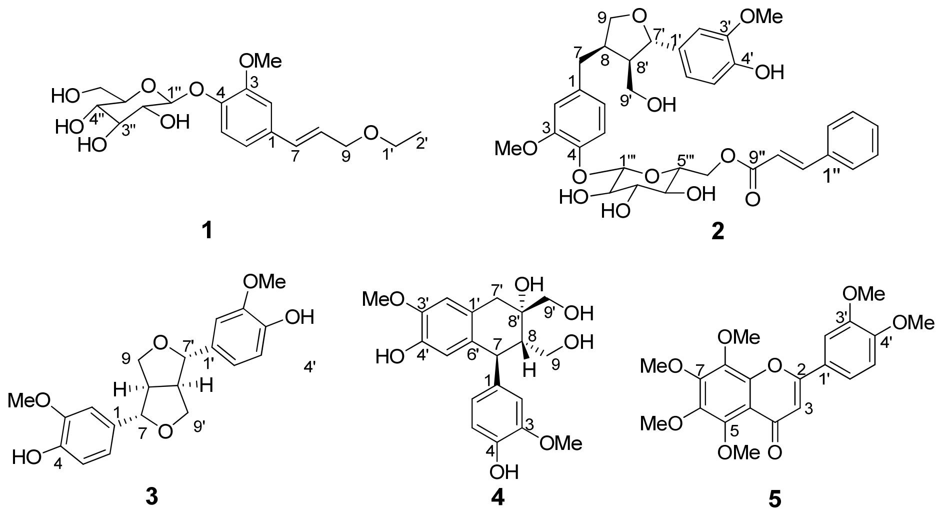

**Figure S2.** Main HMBC Correlations of Compounds **1** and **2**.

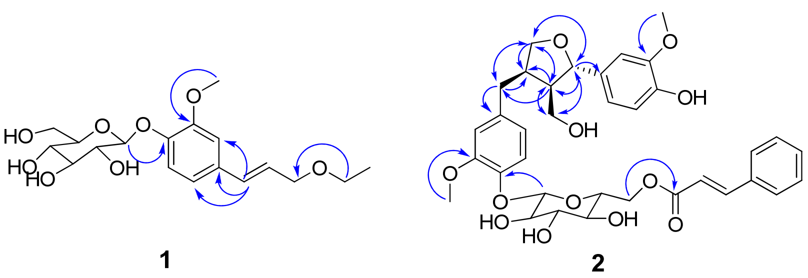

**Figure S3.** Main NOESY Correlations of Compound **2**.

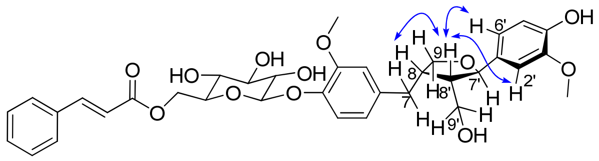

**Figure S4.** The UV Spectrum of Compound 1 in $CH_3OH$.

Company: Print Date: 2012-12-18 21:27:00

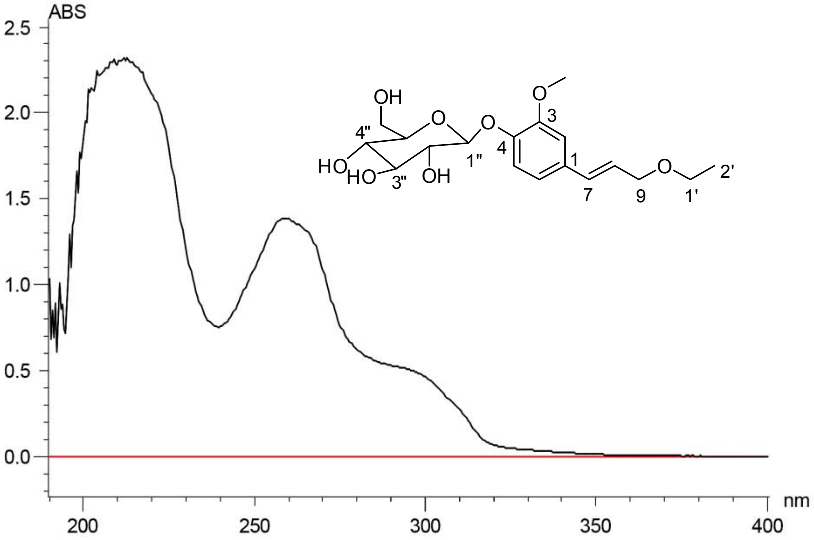


File Name: qh-hsx-10(3).wscn
Date: 2012-12-18 21:23:04
Operator:
Name: Data 9
Comment:

Model: DB-20R
ROM Ver: 03
Manuf No: 6618005

<<<<< Instrument Parameters >>>>>

| | |
|---|---|
| Measurement Type: | WL Scan |
| Data Mode: | ABS |
| Start WL: | 400.0 |
| End WL: | 190.0 |
| Scan Speed: | 400 nm/min |
| Sampling Interval: | 0.5 |
| Slit Width: | 1.0 |
| Lamp Change Mode: | Auto |
| Auto Change WL: | 340.0 |
| Baseline: | User 1 |
| Response: | Fast |
| Path Length: | 10.0 |

(ABS values are corrected to 10mm path length)

**Figure S5.** The IR Spectrum of Compound **1.**

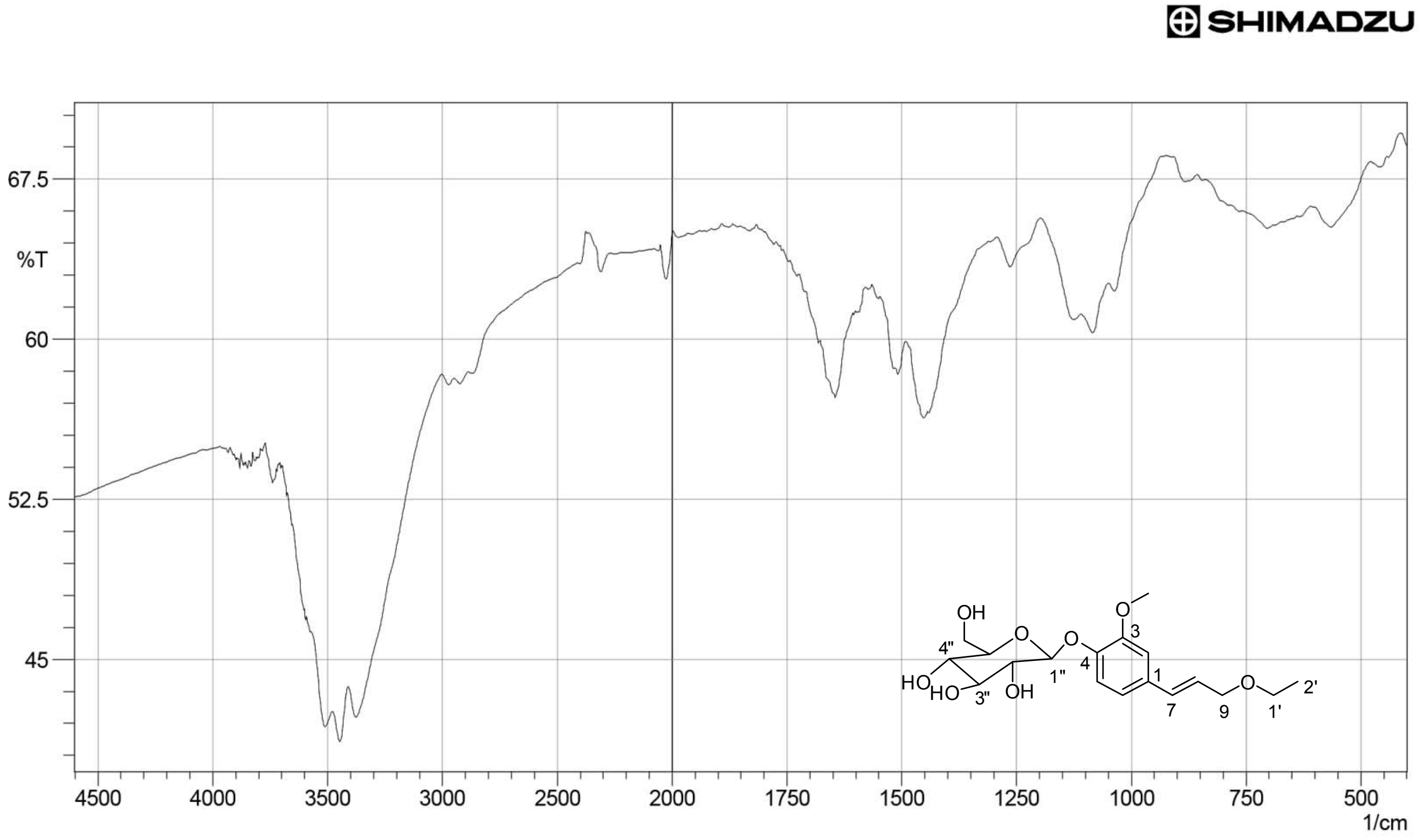

**Figure S6.** The HR-QTF-MS Spectrum of Compound **1**.

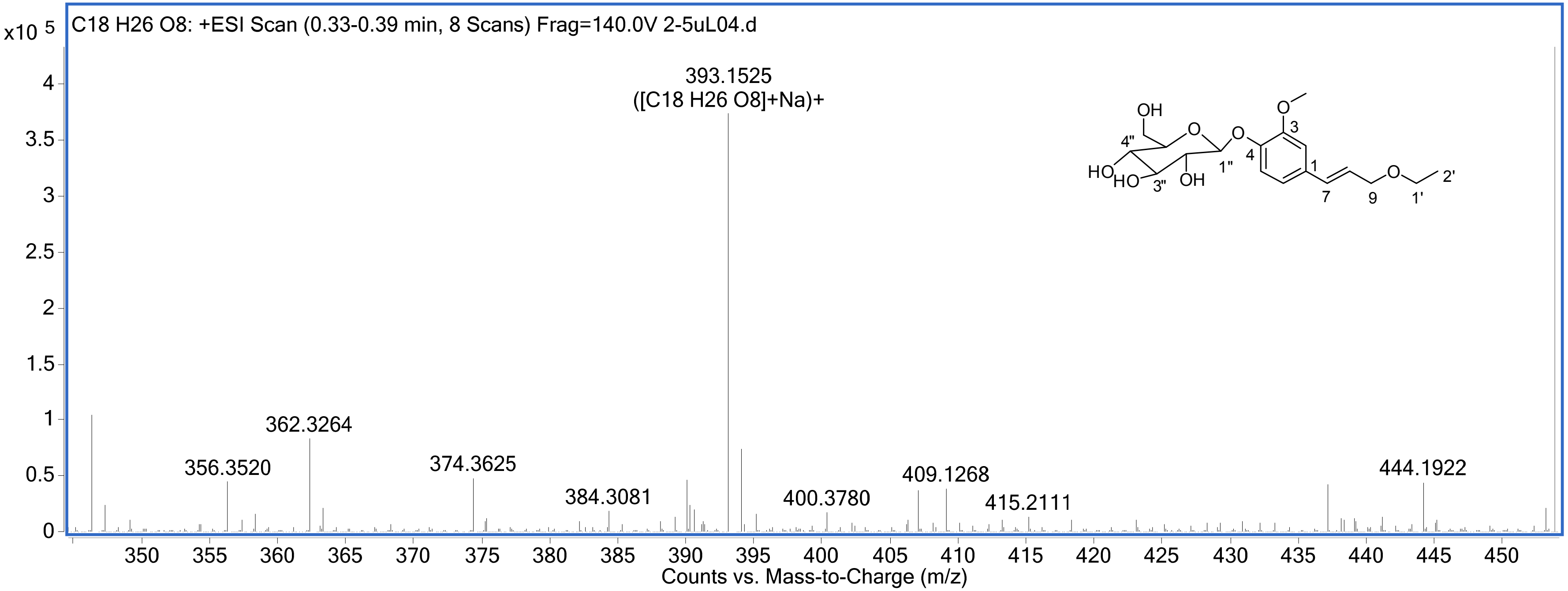

**Figure S7.** The $^{1}$H -NMR Spectrum of Compound 1 in $CD_3OD$.

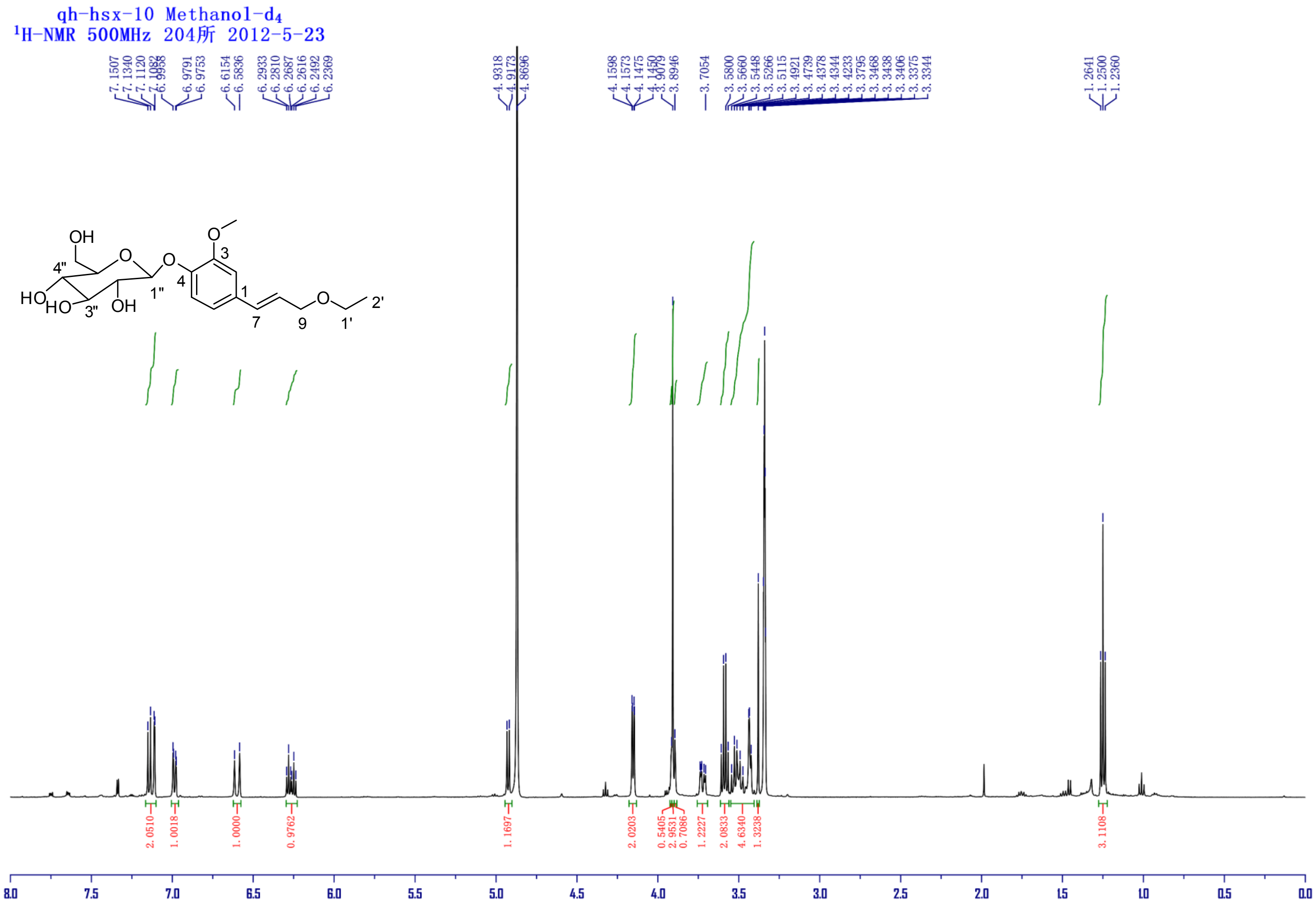

**Figure S8.** The $^{13}$C-NMR Spectrum of Compound 1 in $CD_3OD$.

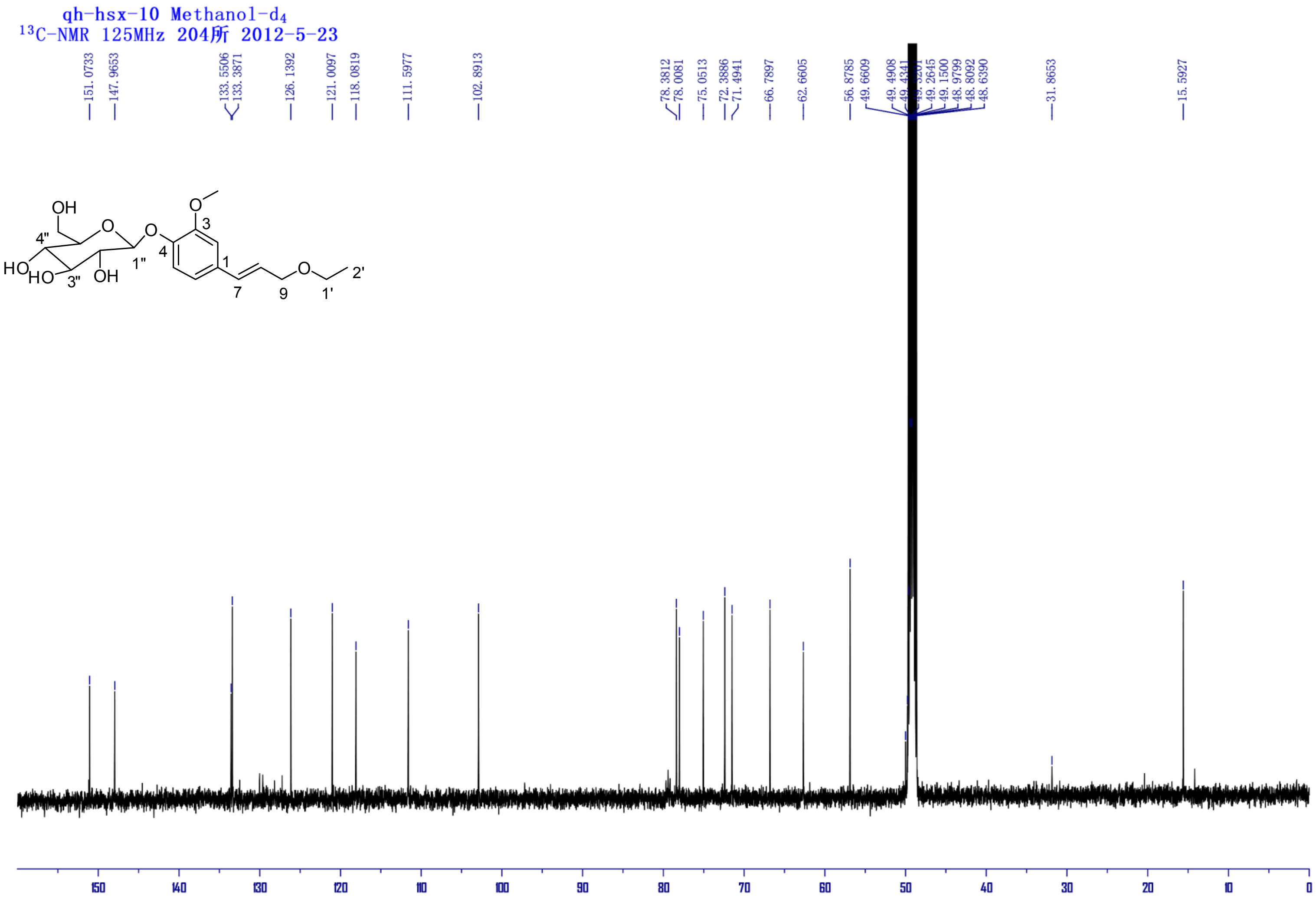

**Figure S9.** The DEPT Spectrum of Compound **1** in $CD_3OD$.

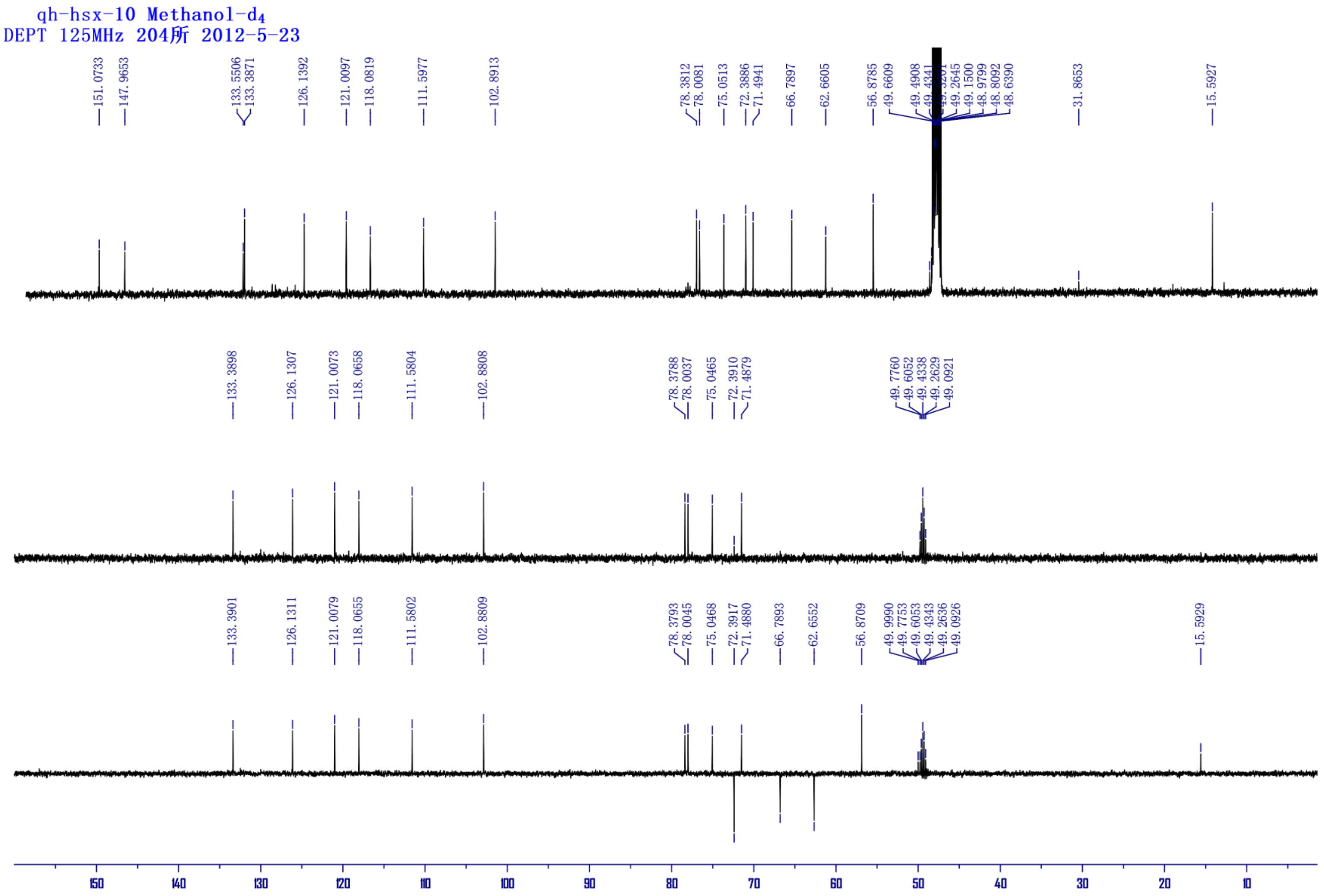

**Figure S10.** The HMQC Spectrum of Compound **1** in $CD_3OD$.

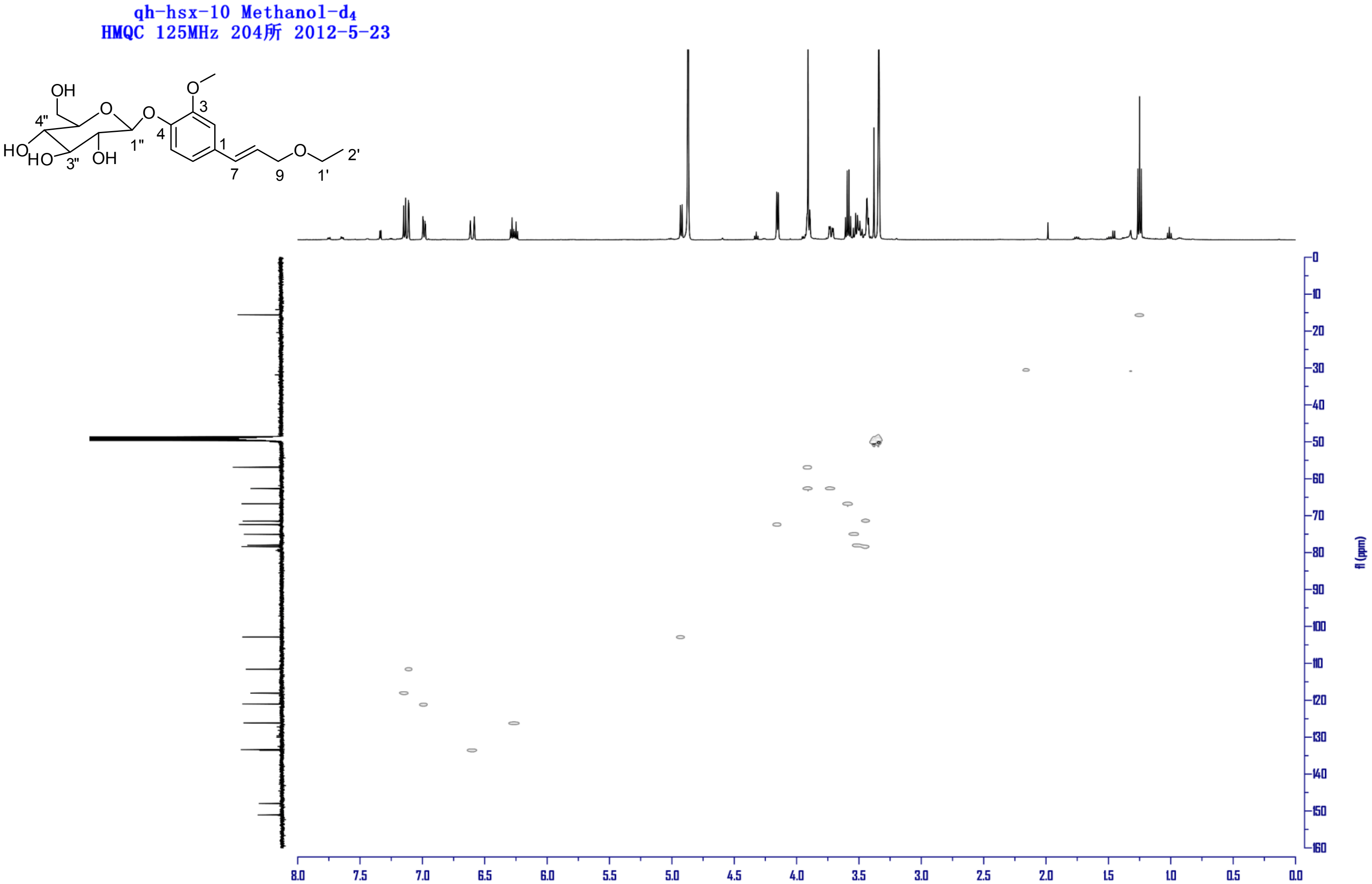

**Figure S11.** The HMBC Spectrum of Compound **1** in $CD_3OD$.

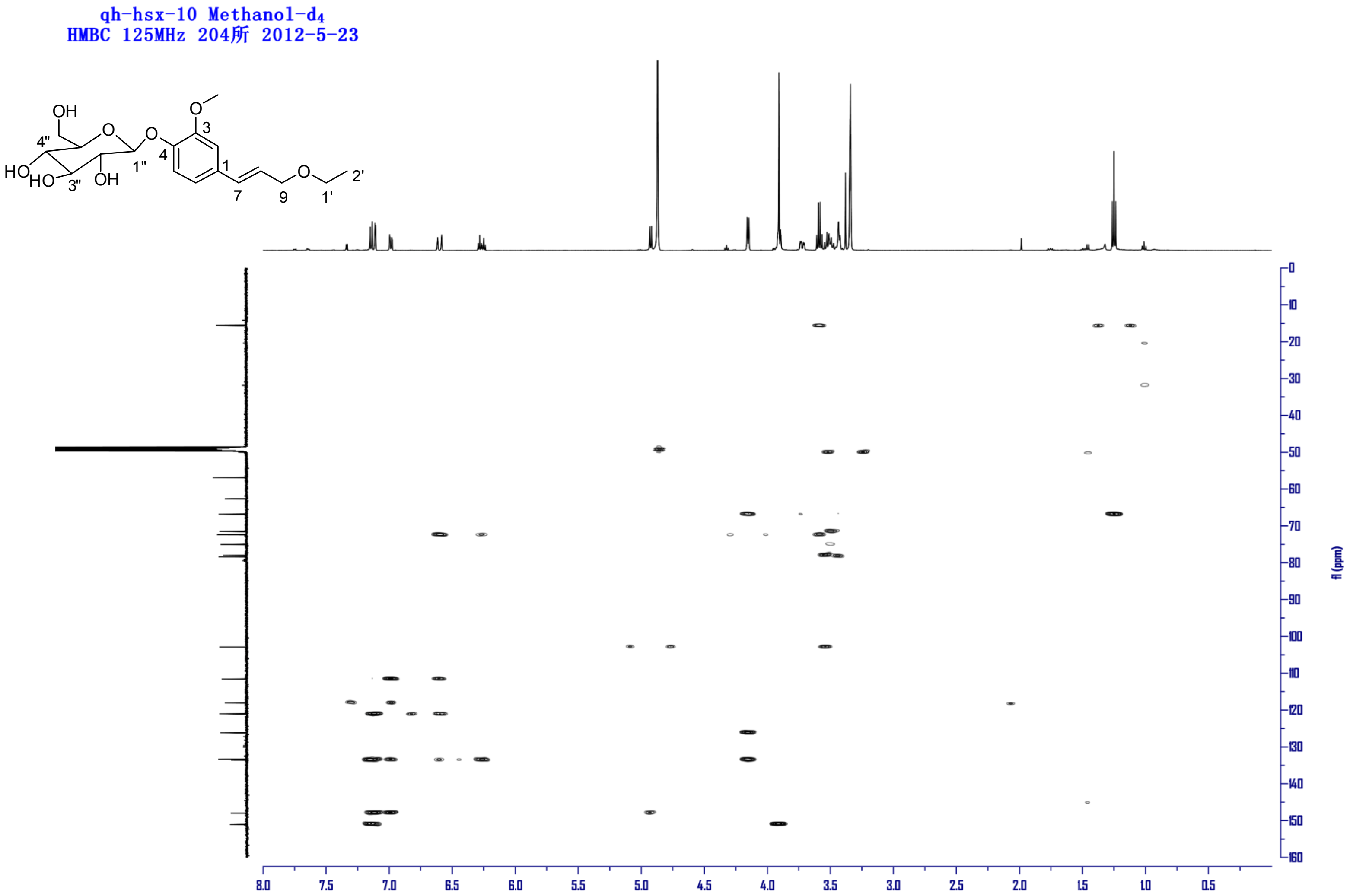

**Figure S12.** The UV Spectrum of Compound **2** in $CH_3OH$.

Company: Print Date: 2012-12-18 23:00:09

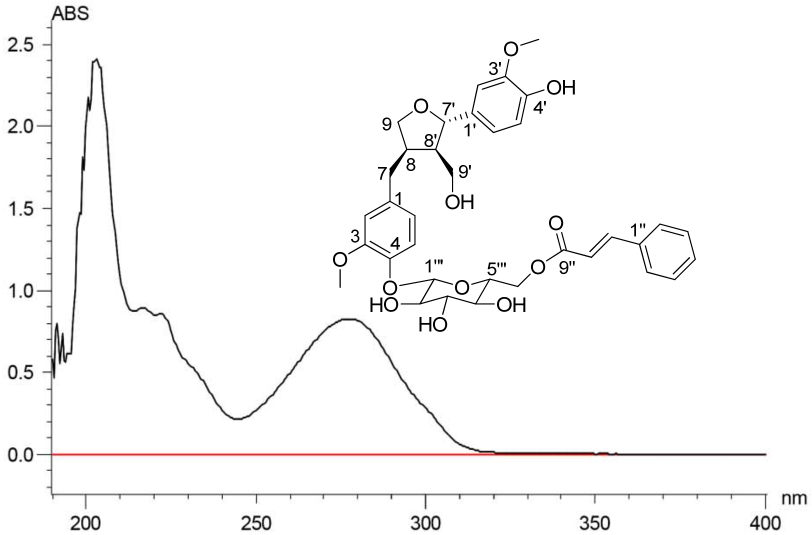


File Name: qh-hsx-66.wscn
Date: 2012-12-18 22:59:26
Operator:
Name: Data 41
Comment:

Model: DB-20R
ROM Ver: 03
Manuf No: 6618005

<<<<< Instrument Parameters >>>>>

| | |
|---|---|
| Measurement Type: | WL Scan |
| Data Mode: | ABS |
| Start WL: | 400.0 |
| End WL: | 190.0 |
| Scan Speed: | 400 nm/min |
| Sampling Interval: | 0.5 |
| Slit Width: | 1.0 |
| Lamp Change Mode: | Auto |
| Auto Change WL: | 340.0 |
| Baseline: | User 1 |
| Response: | Fast |
| Path Length: | 10.0 |

(ABS values are corrected to 10mm path length)

**Figure S13.** The IR Spectrum of Compound **2**.

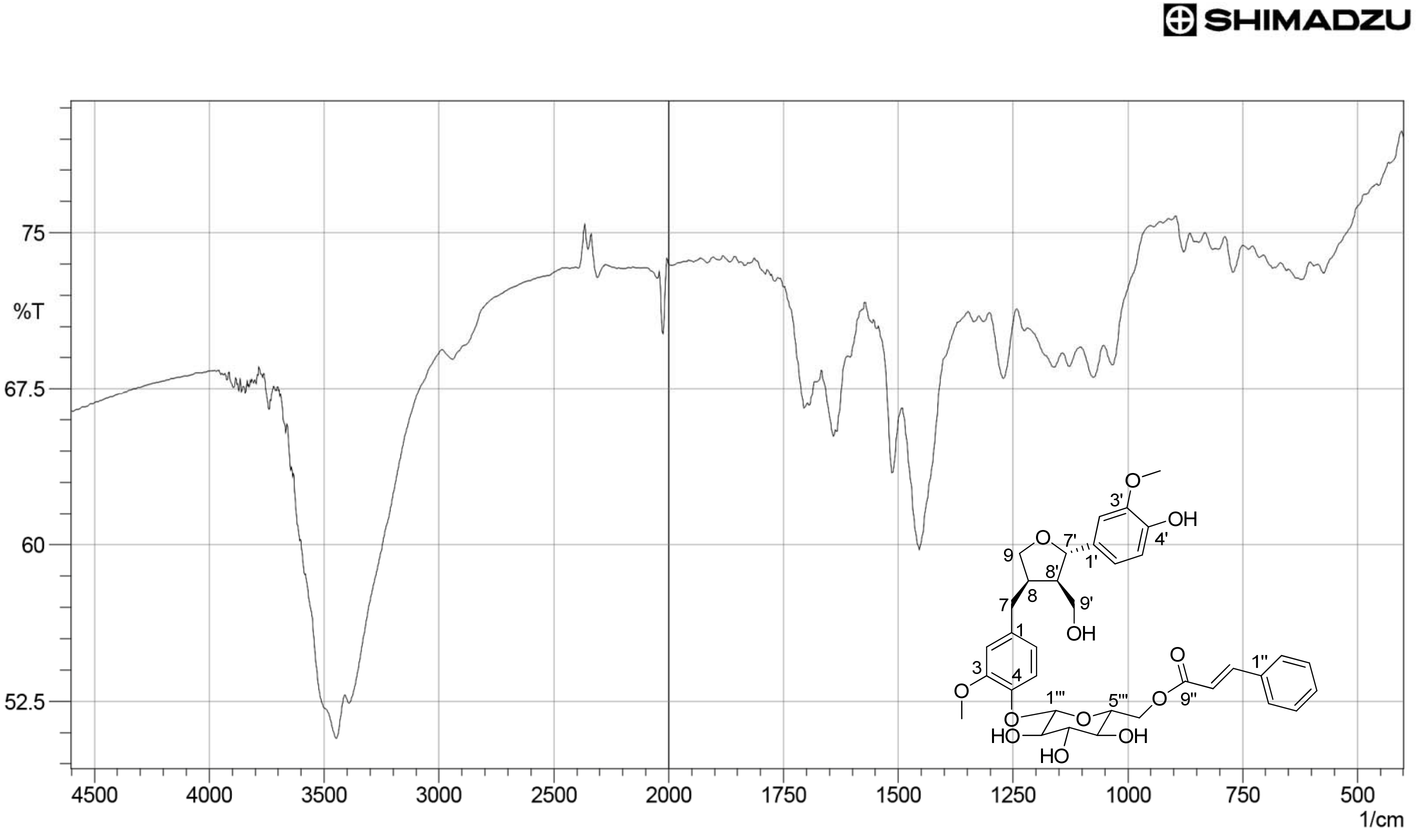

**Figure S14.** The HR-QTF-MS Spectrum of Compound **2**.

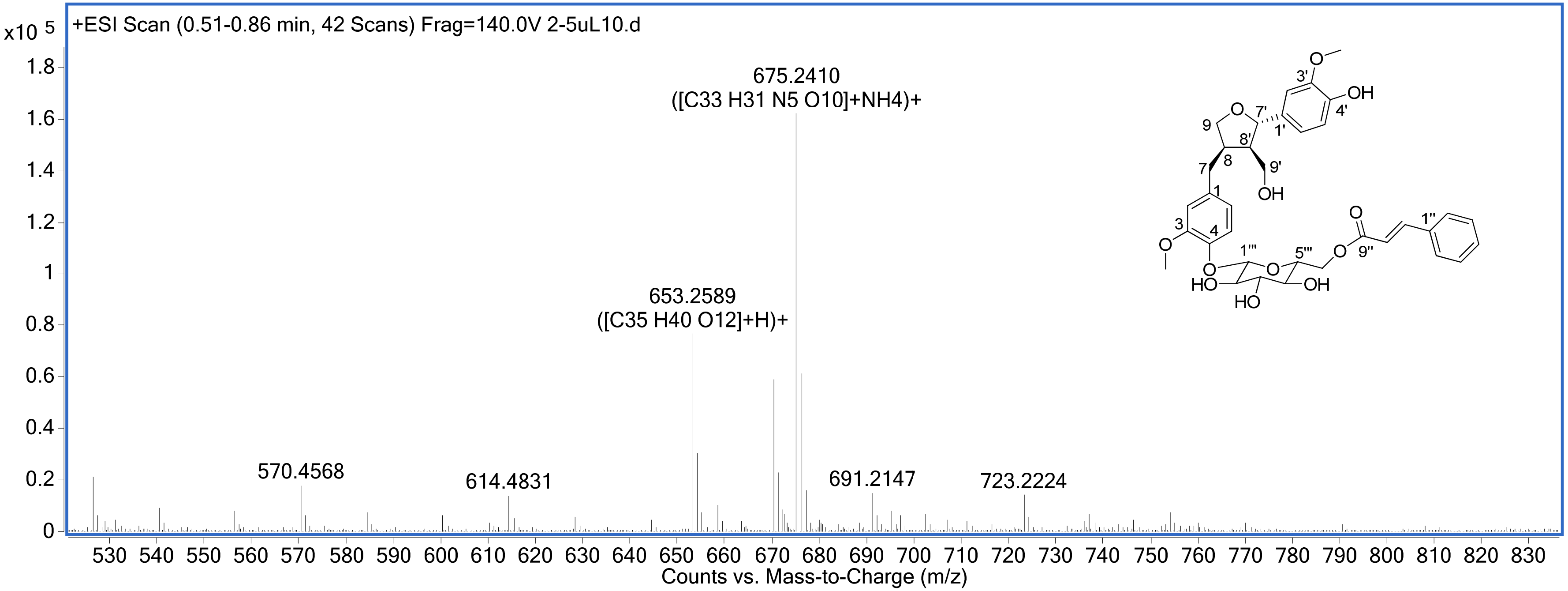

**Figure S15.** The $^{1}$H-NMR Spectrum of Compound **2** in $CD_3OD$.

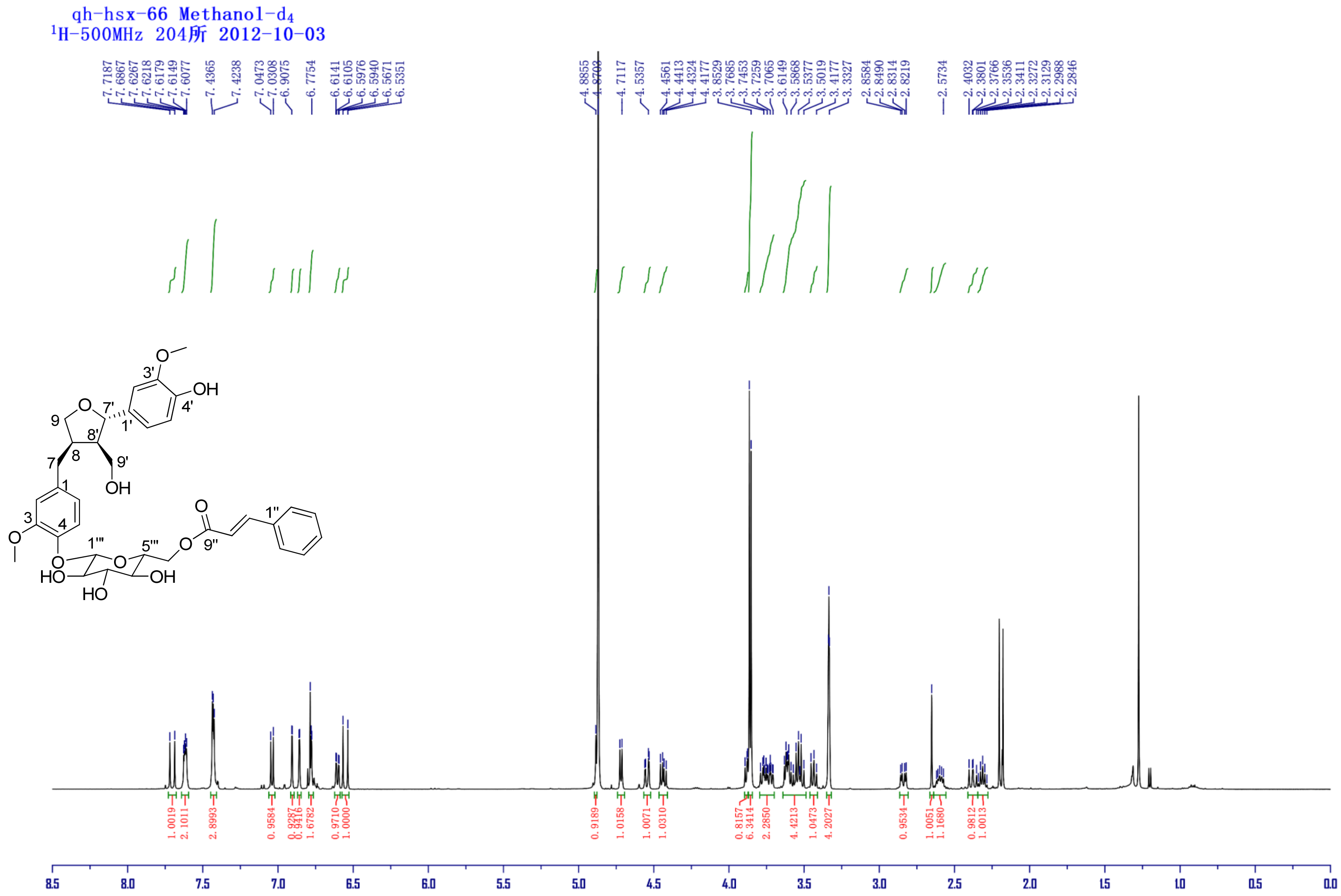

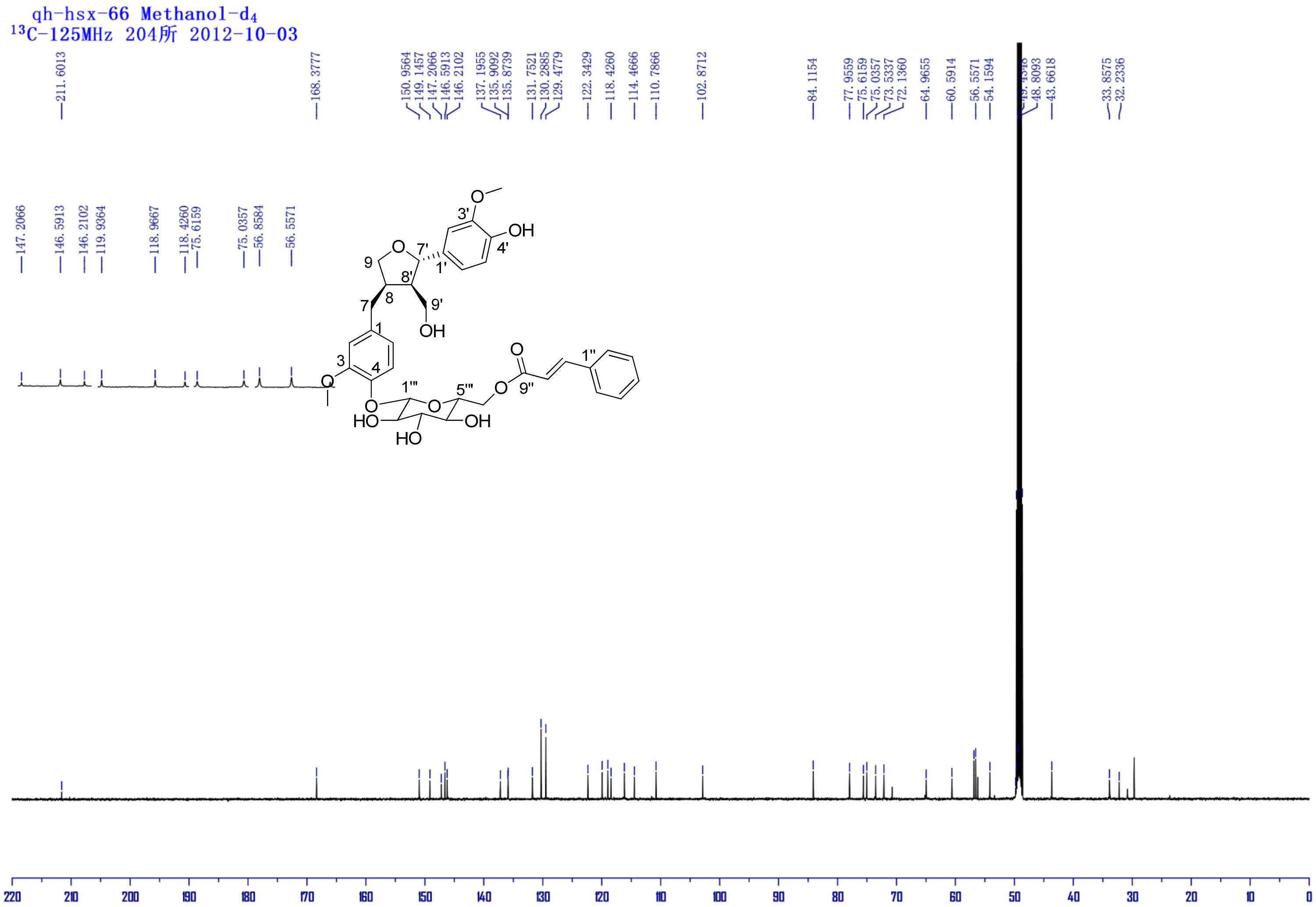


**Figure S16.** The $^{13}$C-NMR Spectrum of Compound **2** in $CD_3OD$.

**Figure S17.** The DEPT Spectrum of Compound **2** in $CD_3OD$.

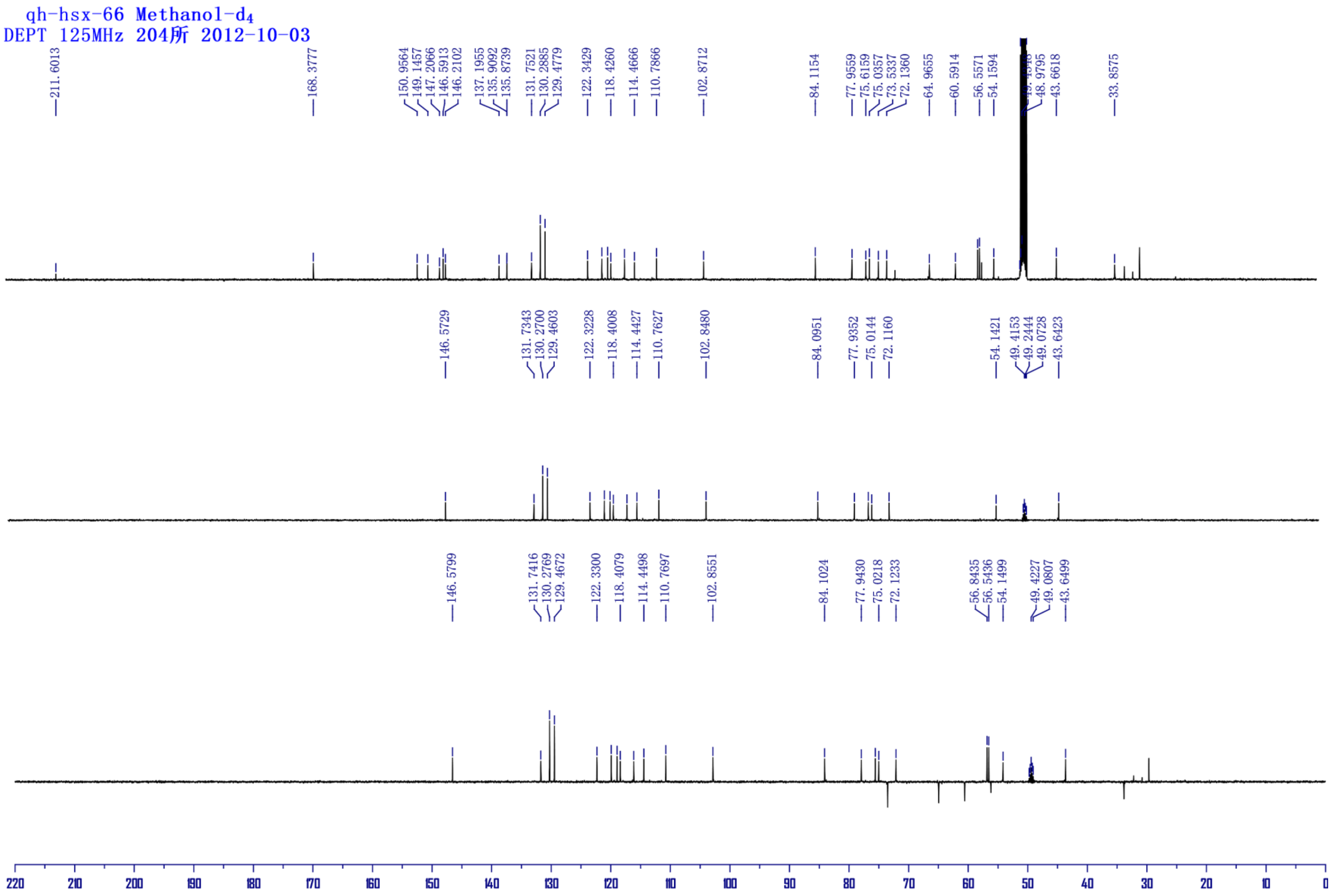

**Figure S18.** The HMQC Spectrum of Compound **2** in $CD_3OD$.

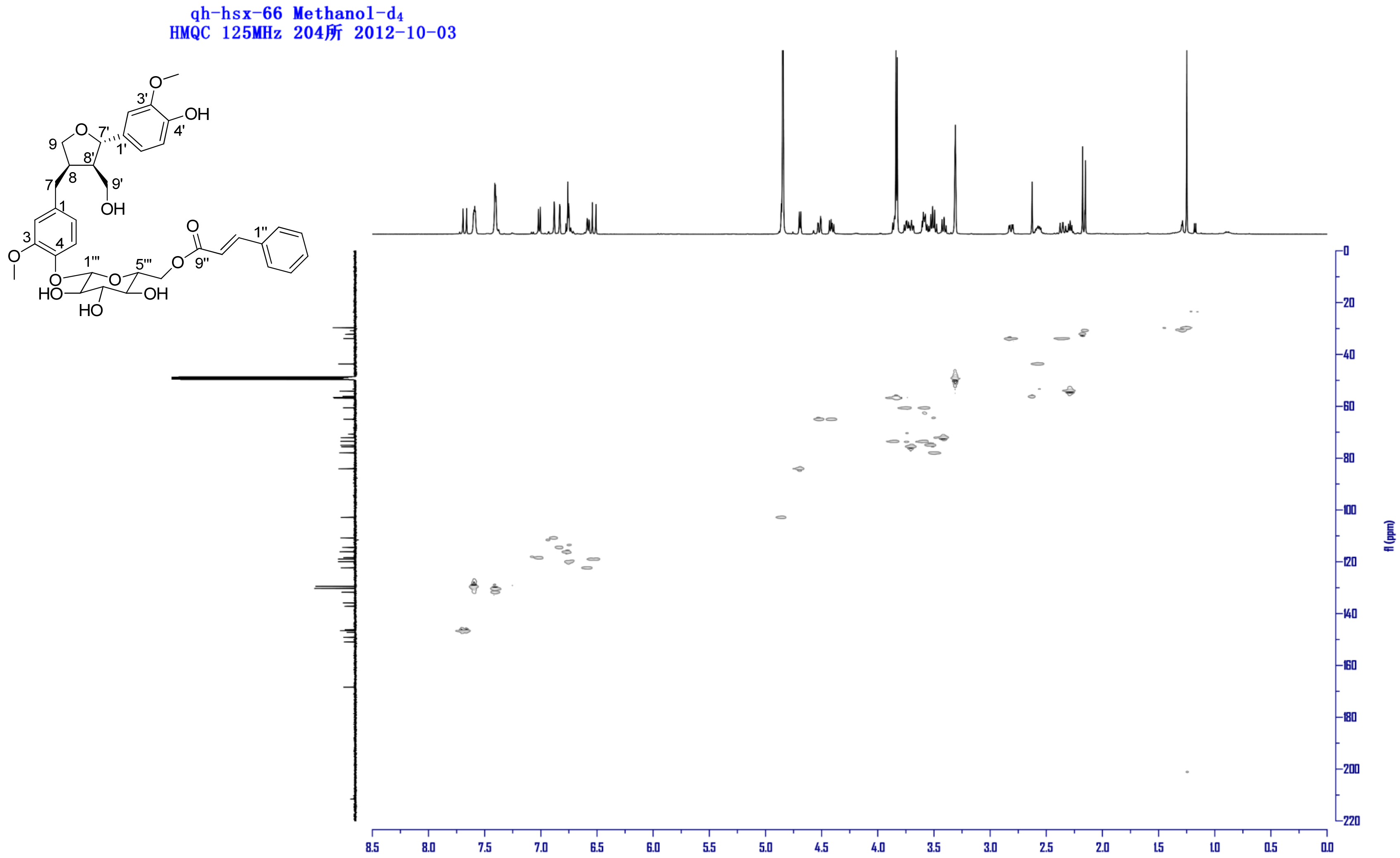

**Figure S19.** The HMBC Spectrum of Compound **2** in $CD_3OD$.

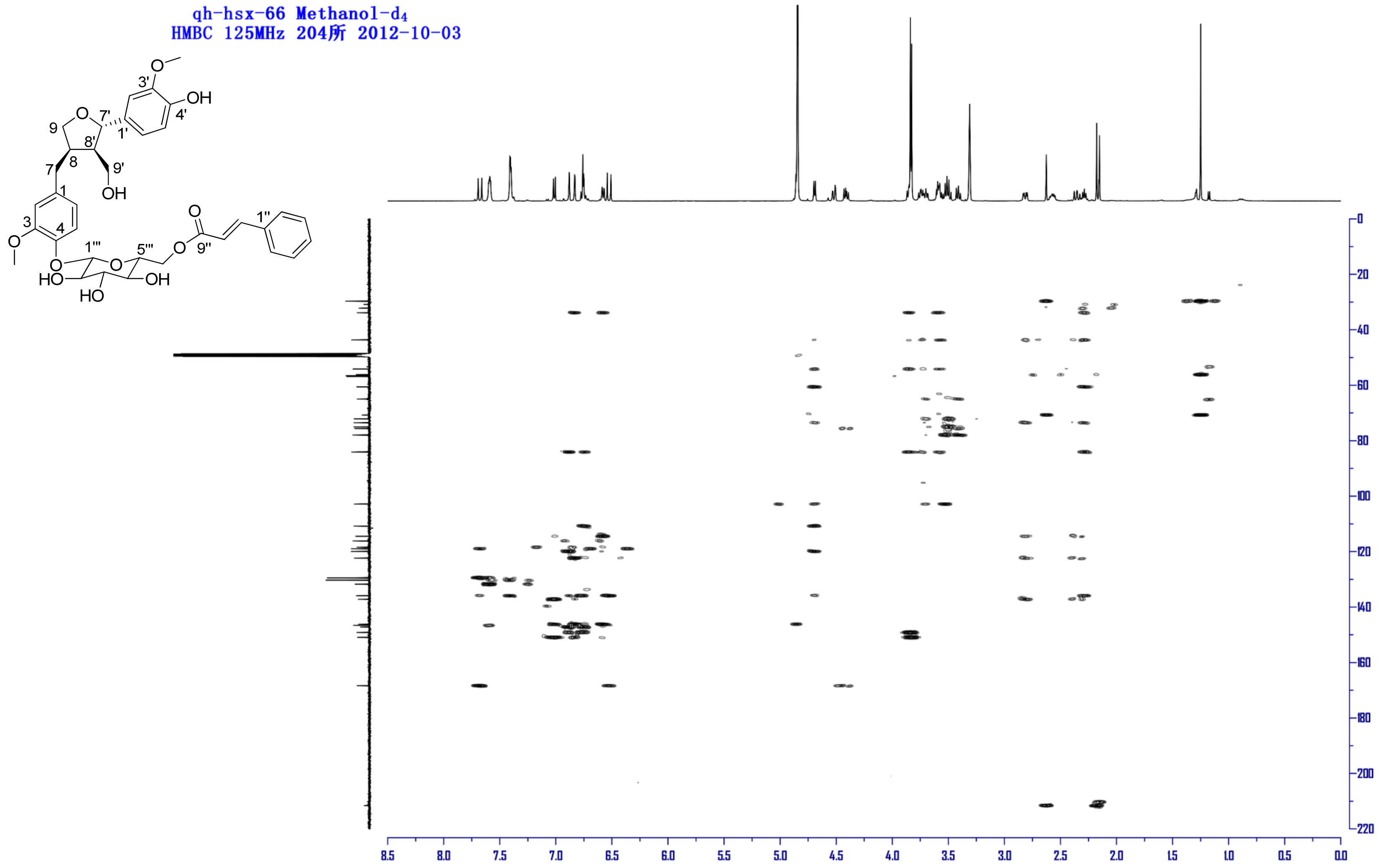

**Figure S20.** The NOESY Spectrum of Compound **2** in $CD_3OD$.

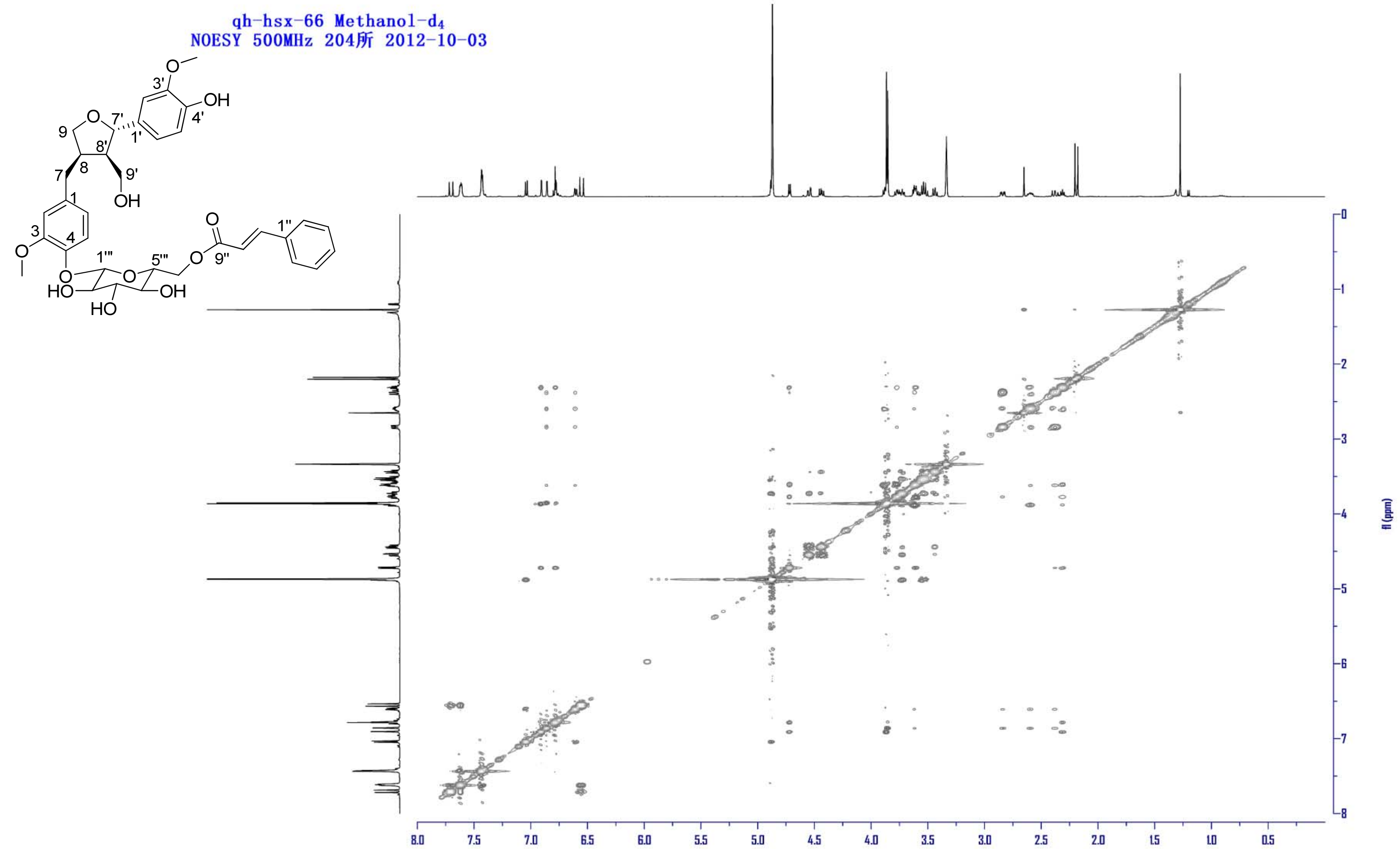

**Figure S21.** The NOESY Spectrum of Compound **2** in $CD_3OD$.

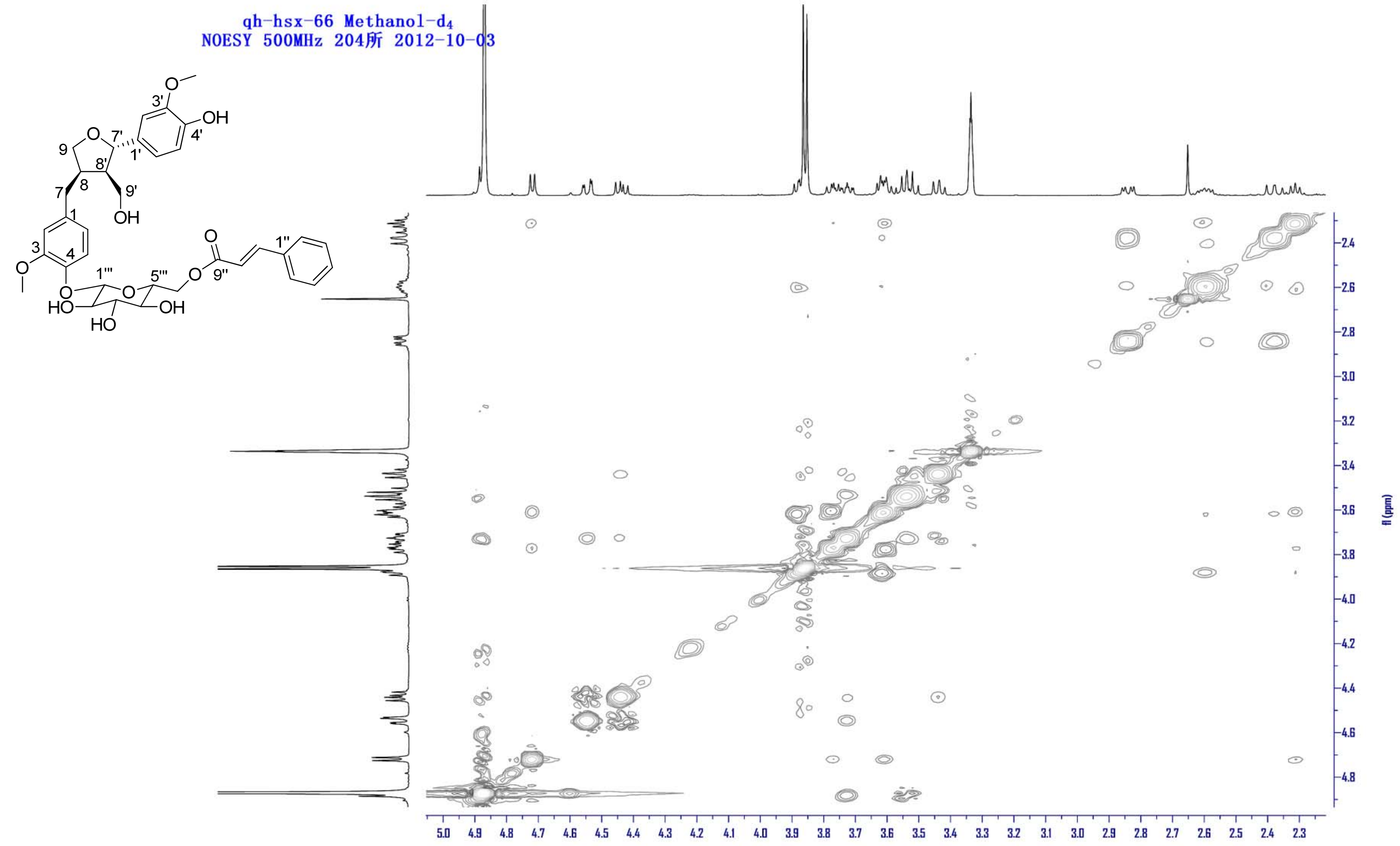

**Figure S22.** The UV Spectrum of Compound **3** in $CH_3OH$.

Company: Print Date: 2012-12-18 21:52:11

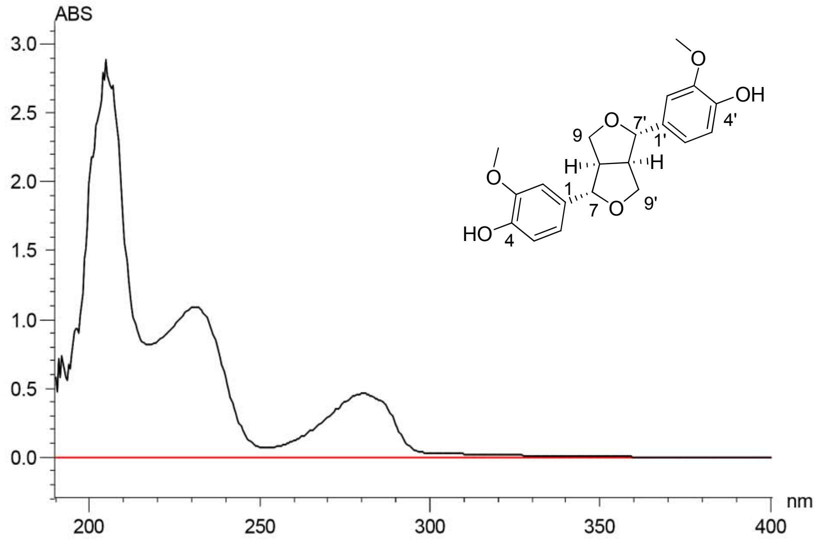


```
File Name:  qh-hsx-19.wscn            Model:      DB-20R
Date:       2012-12-18  21:51:06      ROM Ver:    03
Operator:                             Manuf No:   6618005
Name:       Data 18
Comment:

<<<<< Instrument Parameters >>>>>
Measurement Type:   WL Scan
Data Mode:          ABS
Start WL:           400.0
End WL:             190.0
Scan Speed:         400 nm/min
Sampling Interval:  0.5
Slit Width:         1.0
Lamp Change Mode:   Auto
Auto Change WL:     340.0
Baseline:           User 1
Response:           Fast
Path Length:        10.0
(ABS values are corrected to 10mm path length)
```

**Figure S23.** The IR Spectrum of Compound **3**.

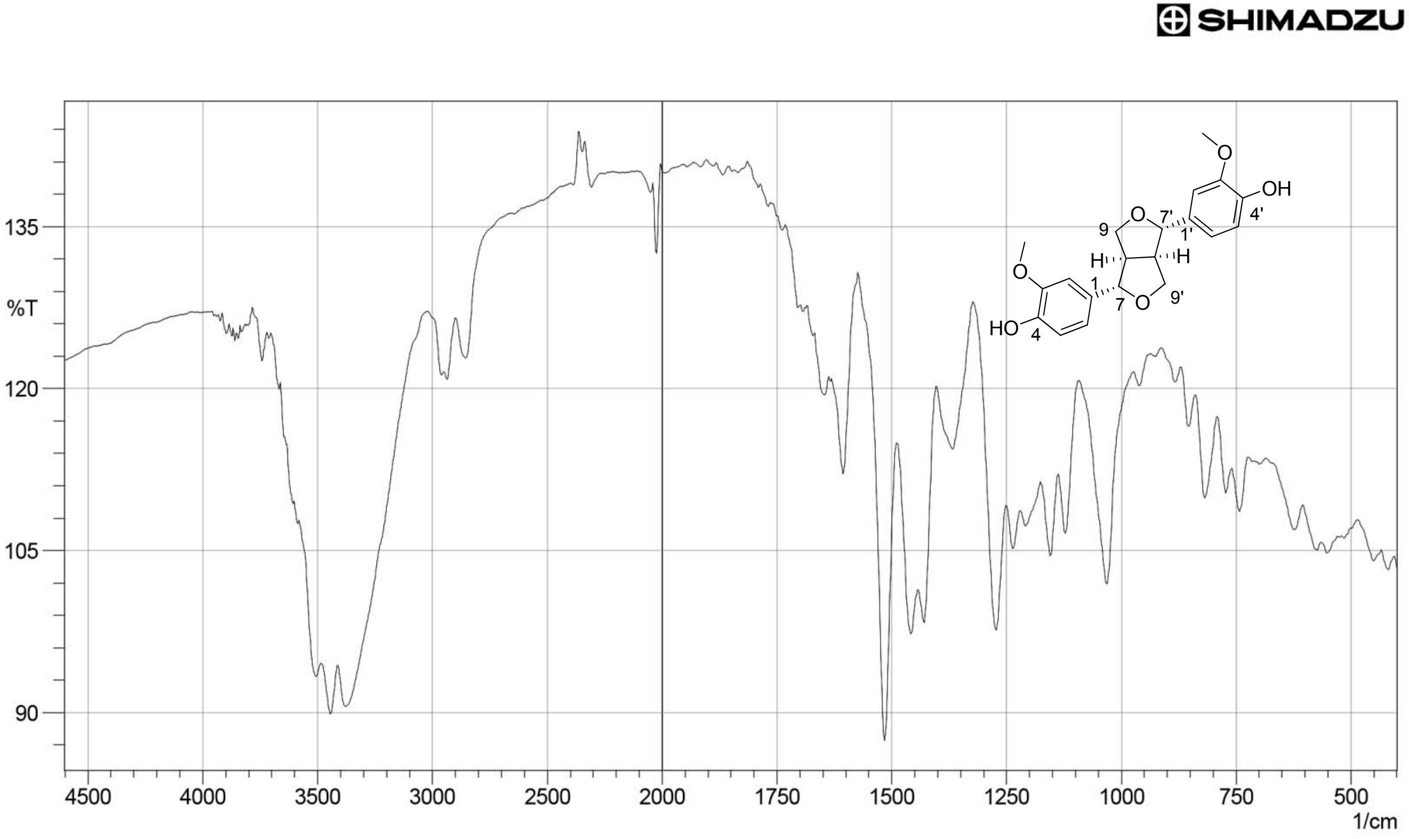

**Figure S24.** The MS Spectrum of Compound **3**.

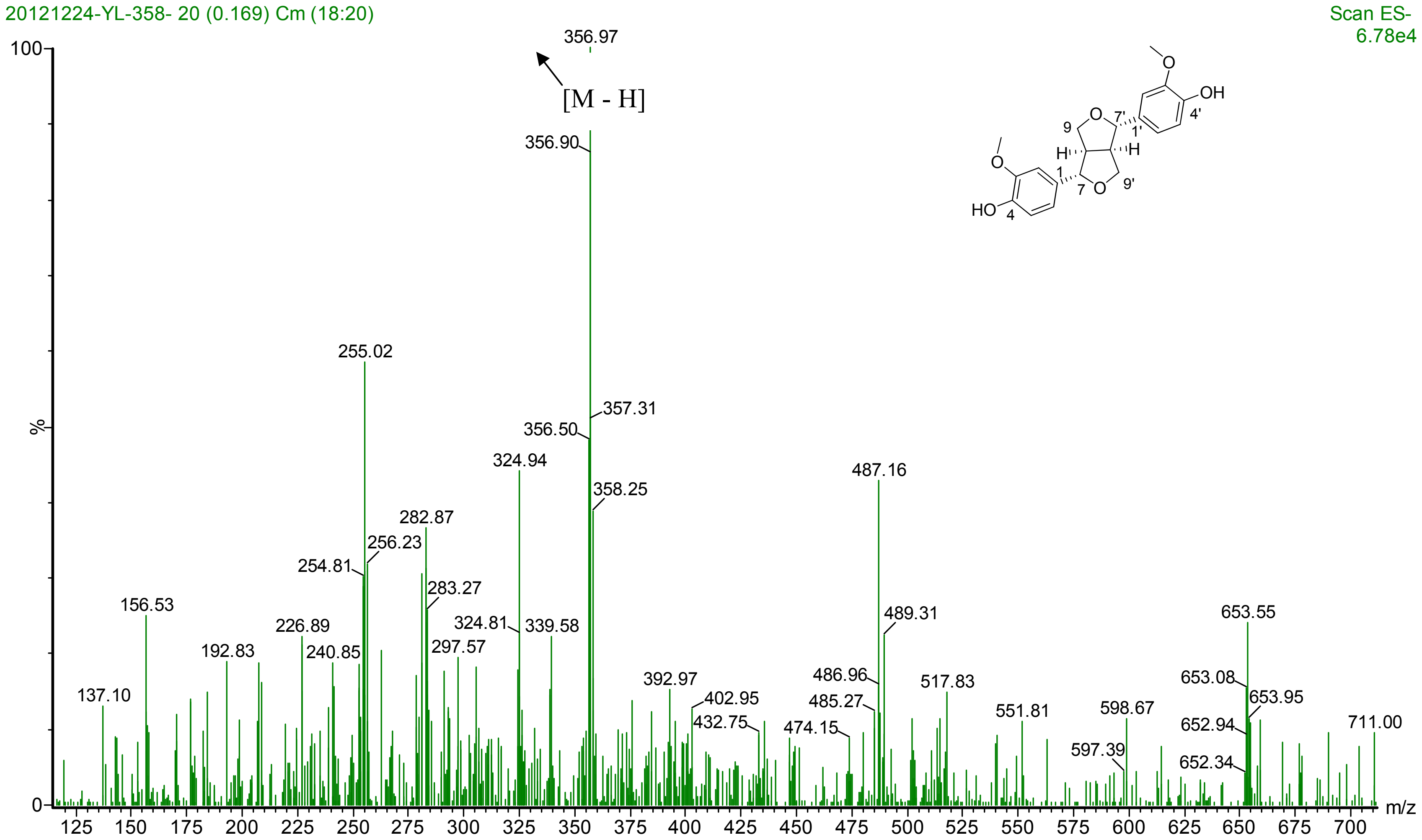

**Figure S25.** The $^{1}$H-NMR Spectrum of Compound **3** in $Me_2CO{-}d_6$.

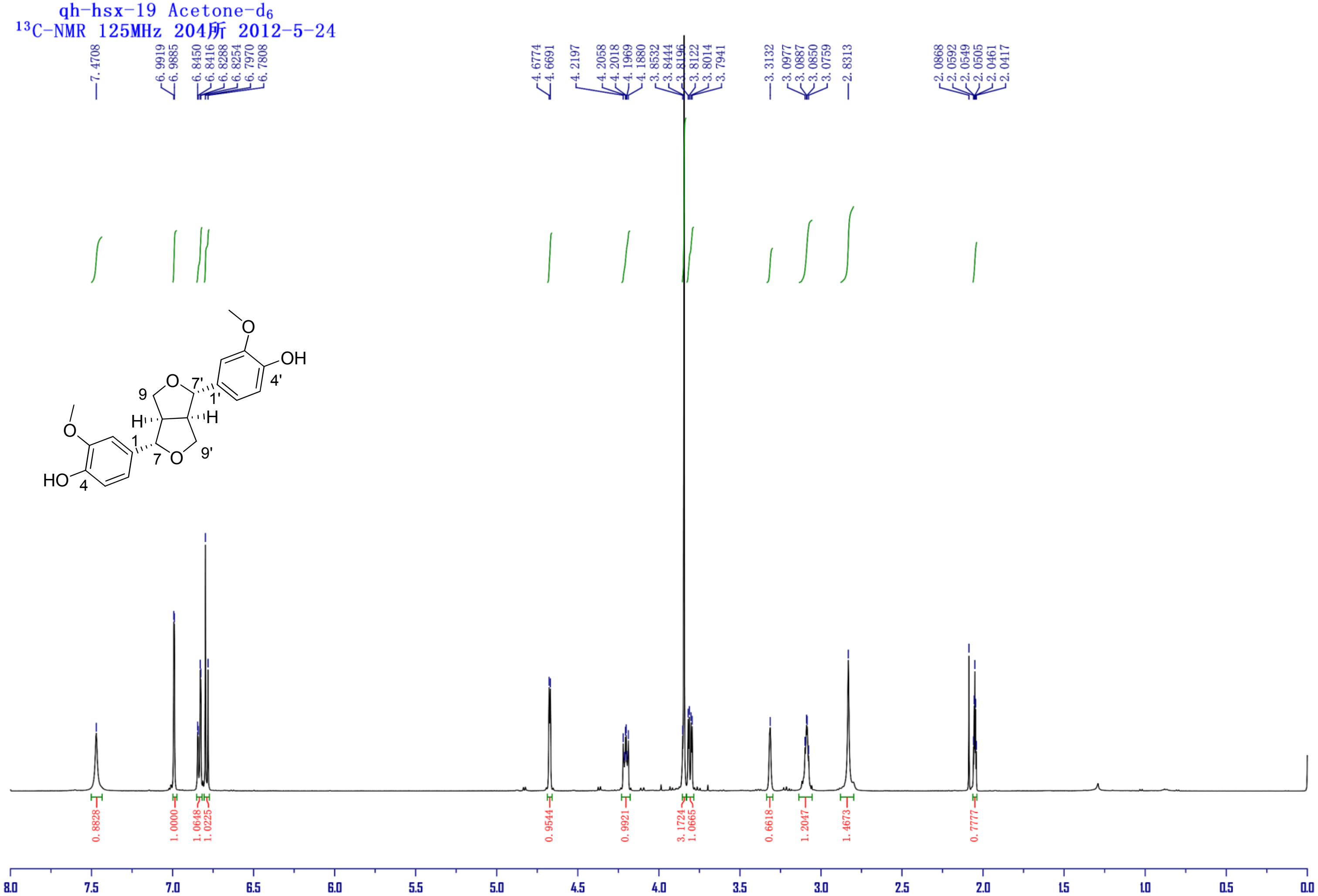

**Figure S26.** The $^{13}$C-NMR Spectrum of Compound **3** in $Me_2CO$–$d_6$.

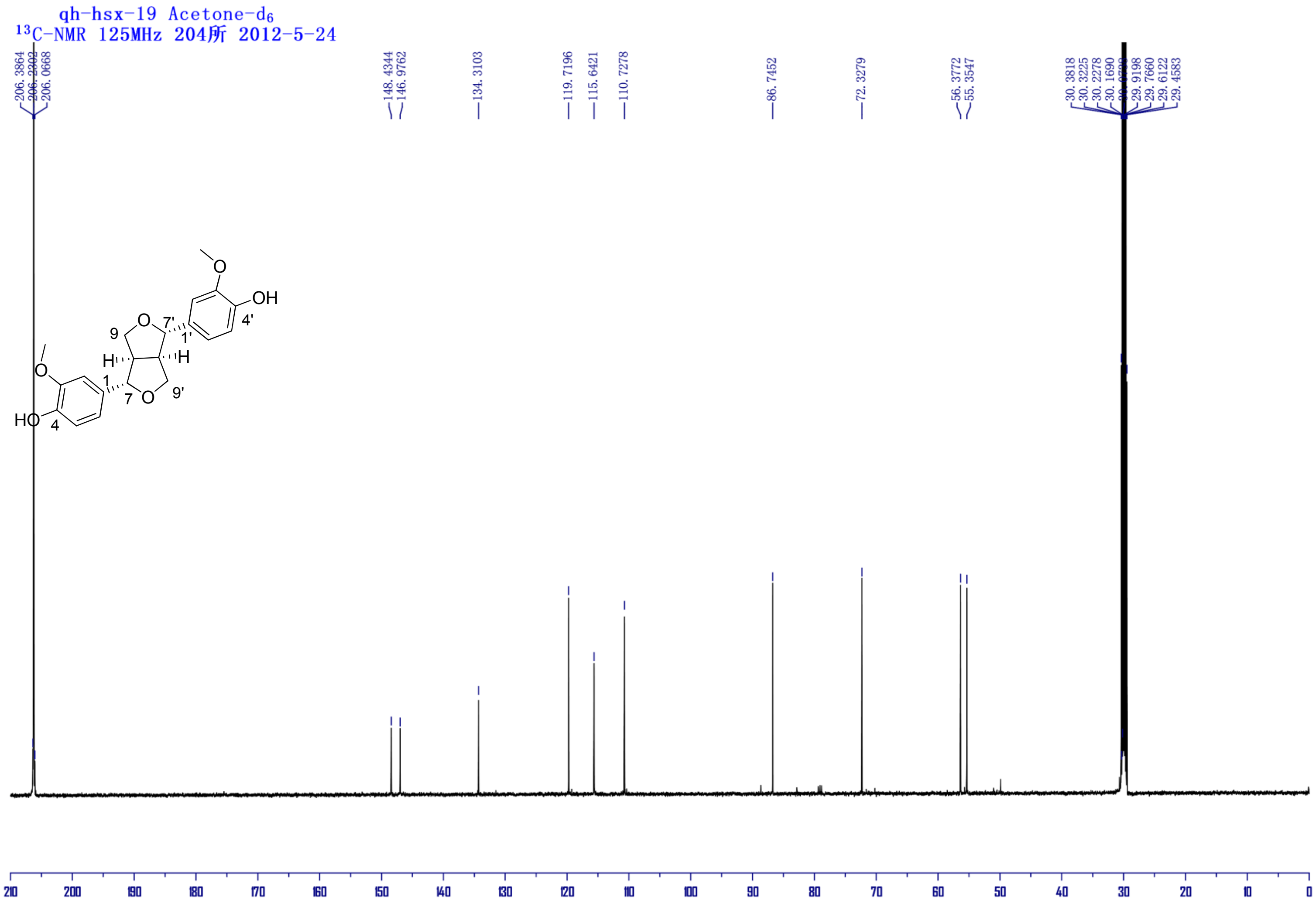

**Figure S27.** The UV Spectrum of Compound **4** in $CH_3OH$.

Company: Print Date: 2012-12-18 22:35:21

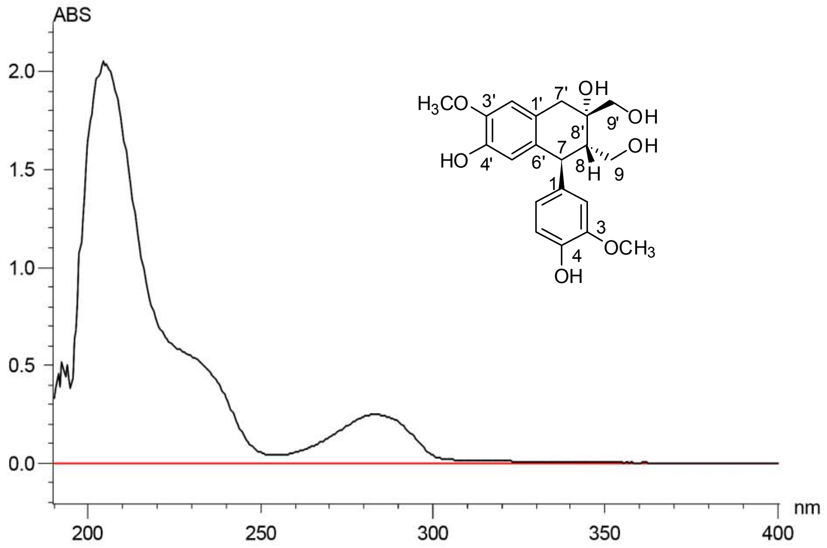


File Name: qh-hsx-39.wscn
Date: 2012-12-18 22:32:35
Operator:
Name: Data 31
Comment:

Model: DB-20R
ROM Ver: 03
Manuf No: 6618005

<<<<< Instrument Parameters >>>>>
Measurement Type: WL Scan
Data Mode: ABS
Start WL: 400.0
End WL: 190.0
Scan Speed: 400 nm/min
Sampling Interval: 0.5
Slit Width: 1.0
Lamp Change Mode: Auto
Auto Change WL: 340.0
Baseline: User 1
Response: Fast
Path Length: 10.0
(ABS values are corrected to 10mm path length)

**Figure S28.** The IR Spectrum of Compound **4**.

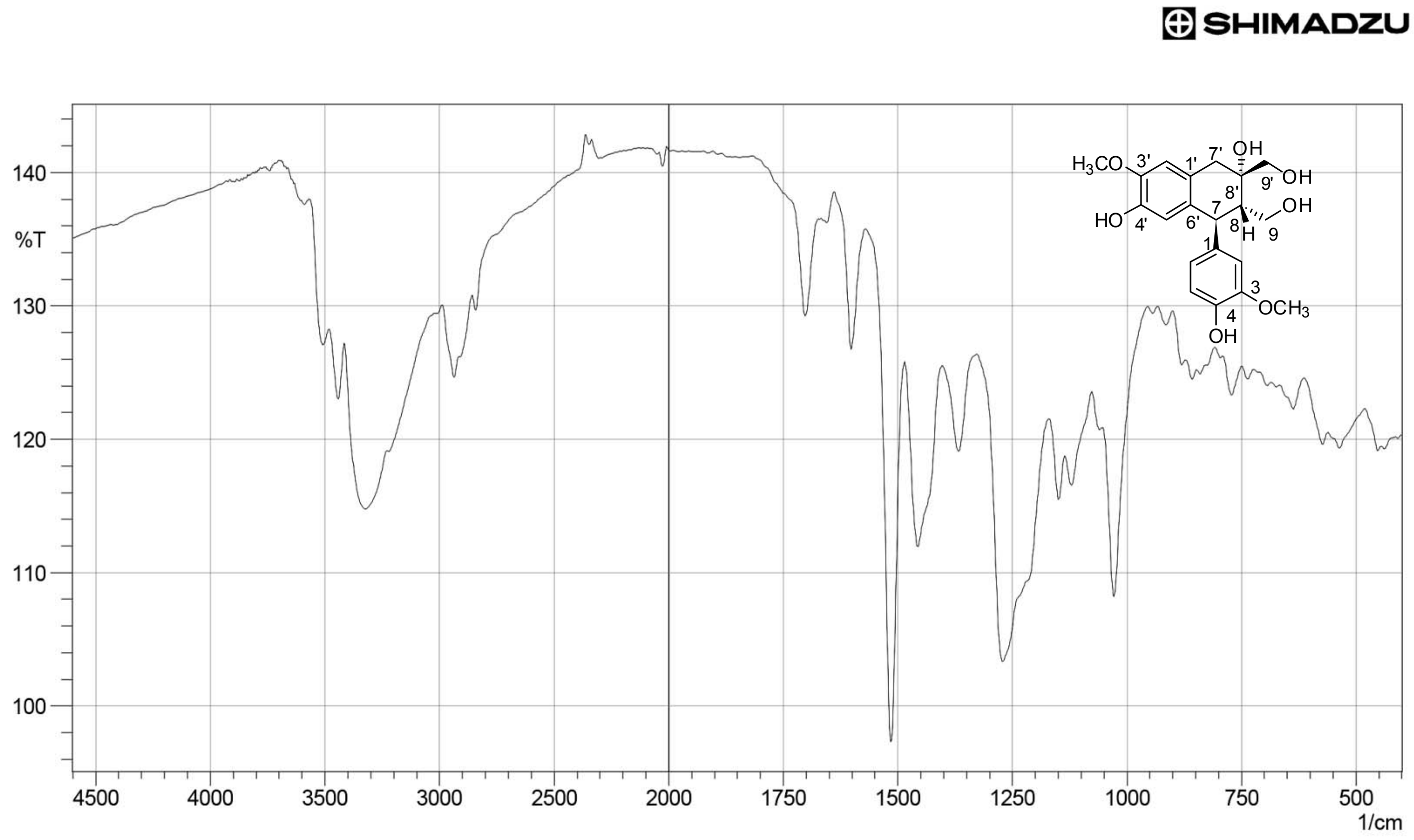

**Figure S29.** The MS Spectrum of Compound **4.**

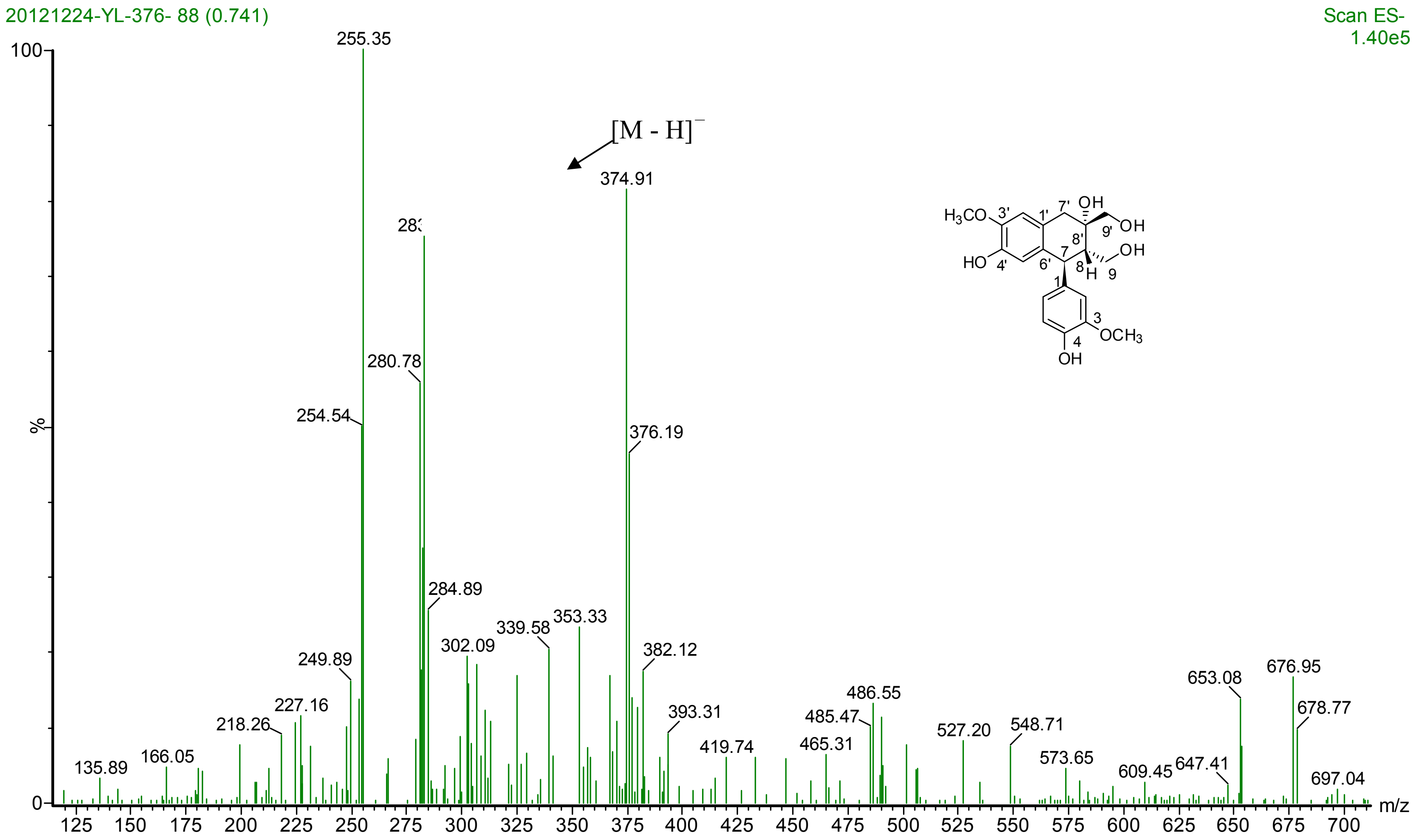

**Figure S30.** The $^{1}$H-NMR Spectrum of Compound **4** in $CD_3OD$.

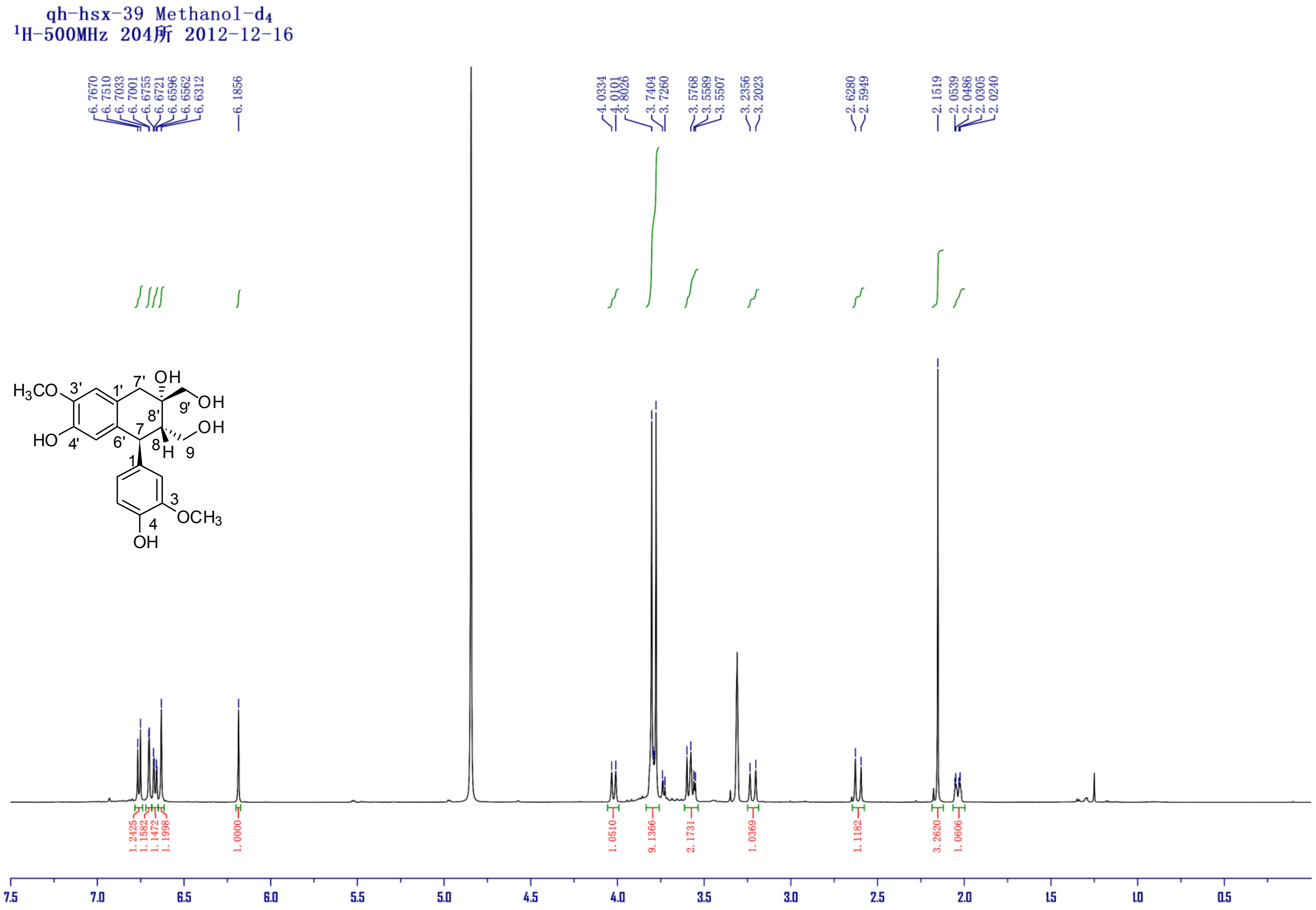

**Figure S31.** The $^{13}$C-NMR Spectrum of Compound **4** in $CD_3OD$.

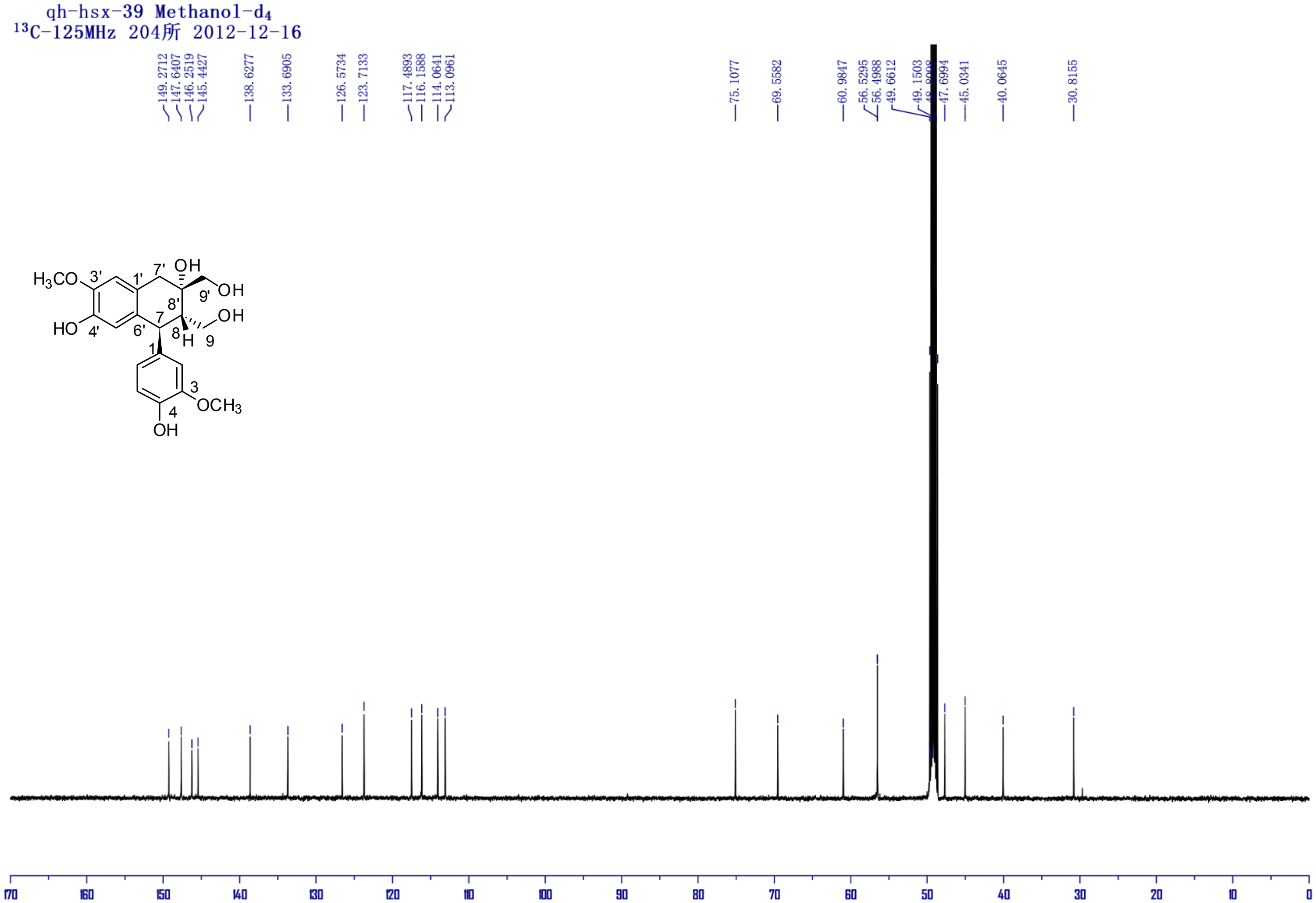

**Figure S32.** The DEPT Spectrum of Compound **4** in $CD_3OD$.

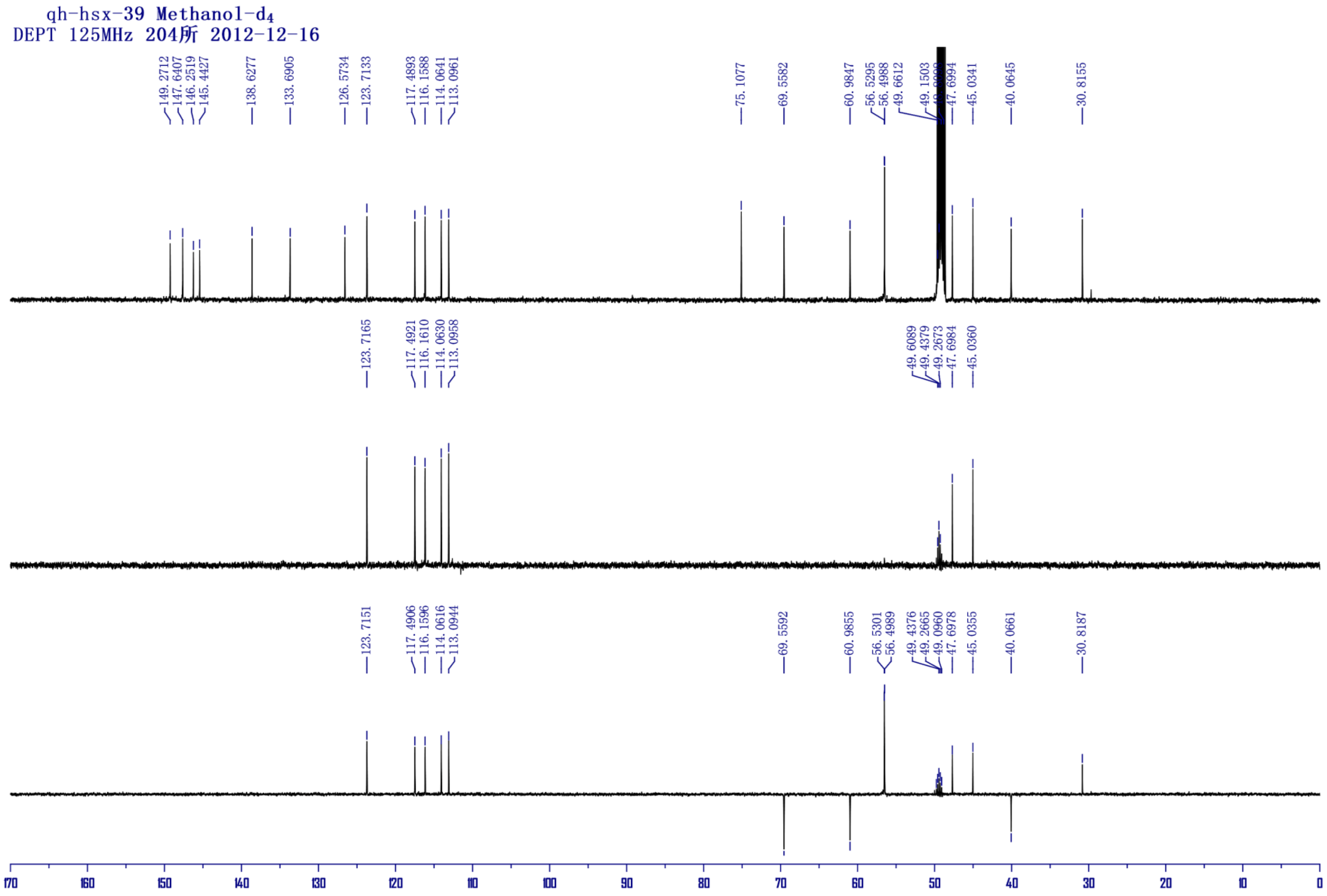

**Figure S33.** The UV Spectrum of Compound **5** in $CH_3OH$.

Company: Print Date: 2012-12-18 23:45:56

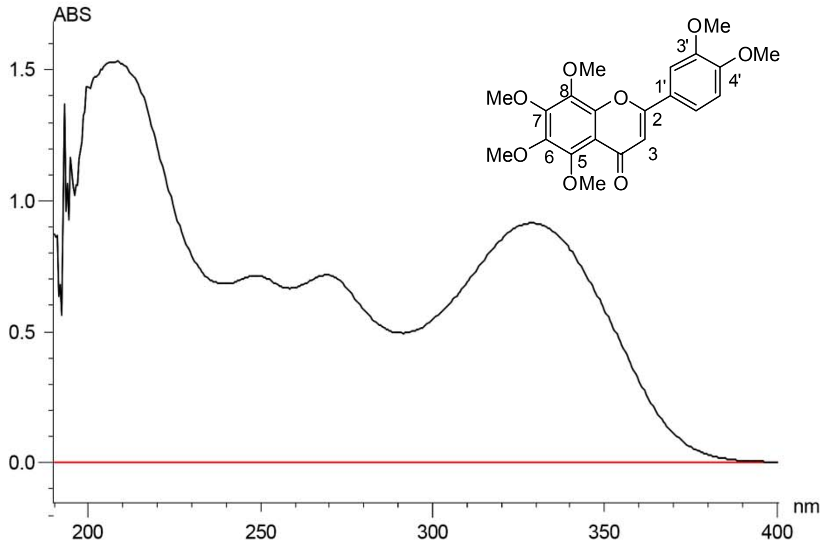


```
File Name:  qh-hsx-75.wscn              Model:      DB-20R
Date:       2012-12-18  23:45:11        ROM Ver:    03
Operator:                               Manuf No:   6618005
Name:       Data 57
Comment:

<<<<< Instrument Parameters >>>>>
Measurement Type:    WL Scan
Data Mode:           ABS
Start WL:            400.0
End WL:              190.0
Scan Speed:          400 nm/min
Sampling Interval:   0.5
Slit Width:          1.0
Lamp Change Mode:    Auto
Auto Change WL:      340.0
Baseline:            User 1
Response:            Fast
Path Length:         10.0
(ABS values are corrected to 10mm path length)
```

**Figure S34.** The IR Spectrum of Compound **5.**

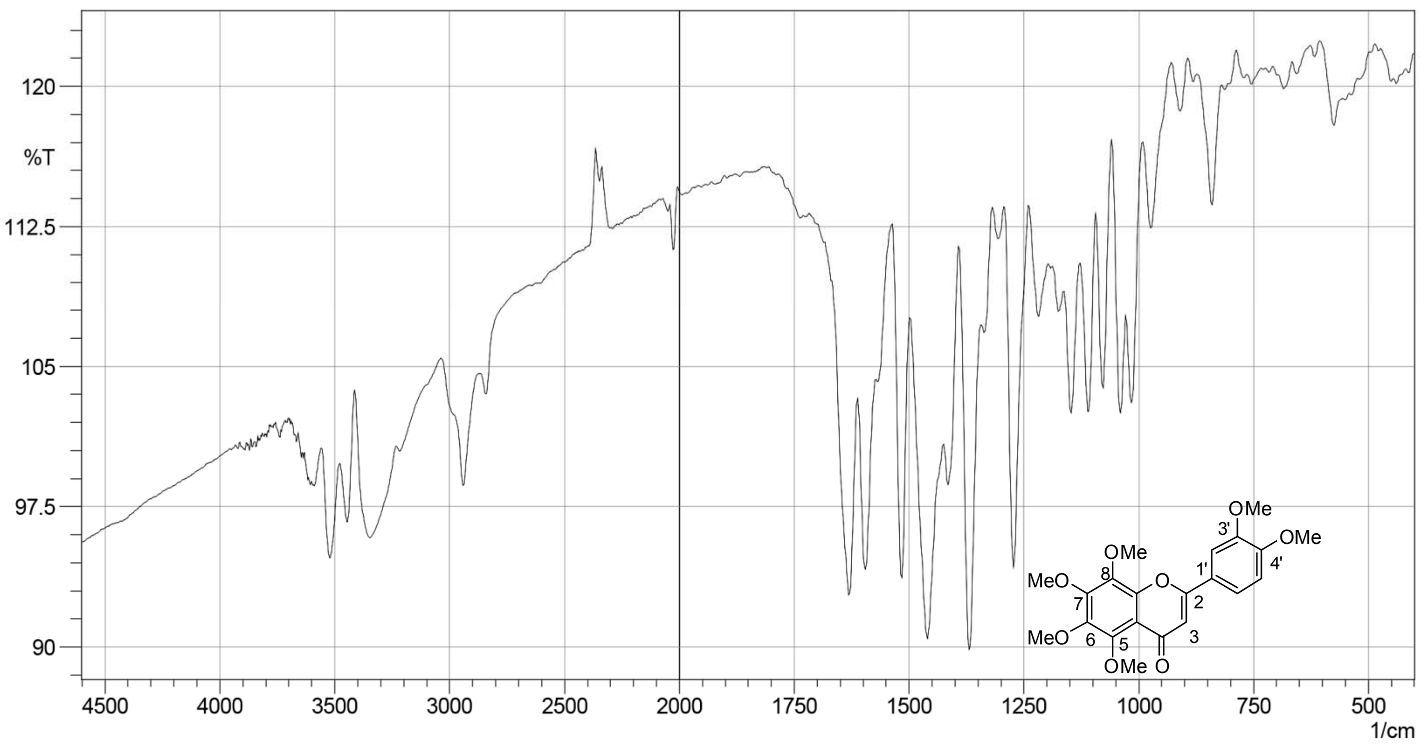

**Figure S35.** The ESIMS Spectrum of Compound **5**.

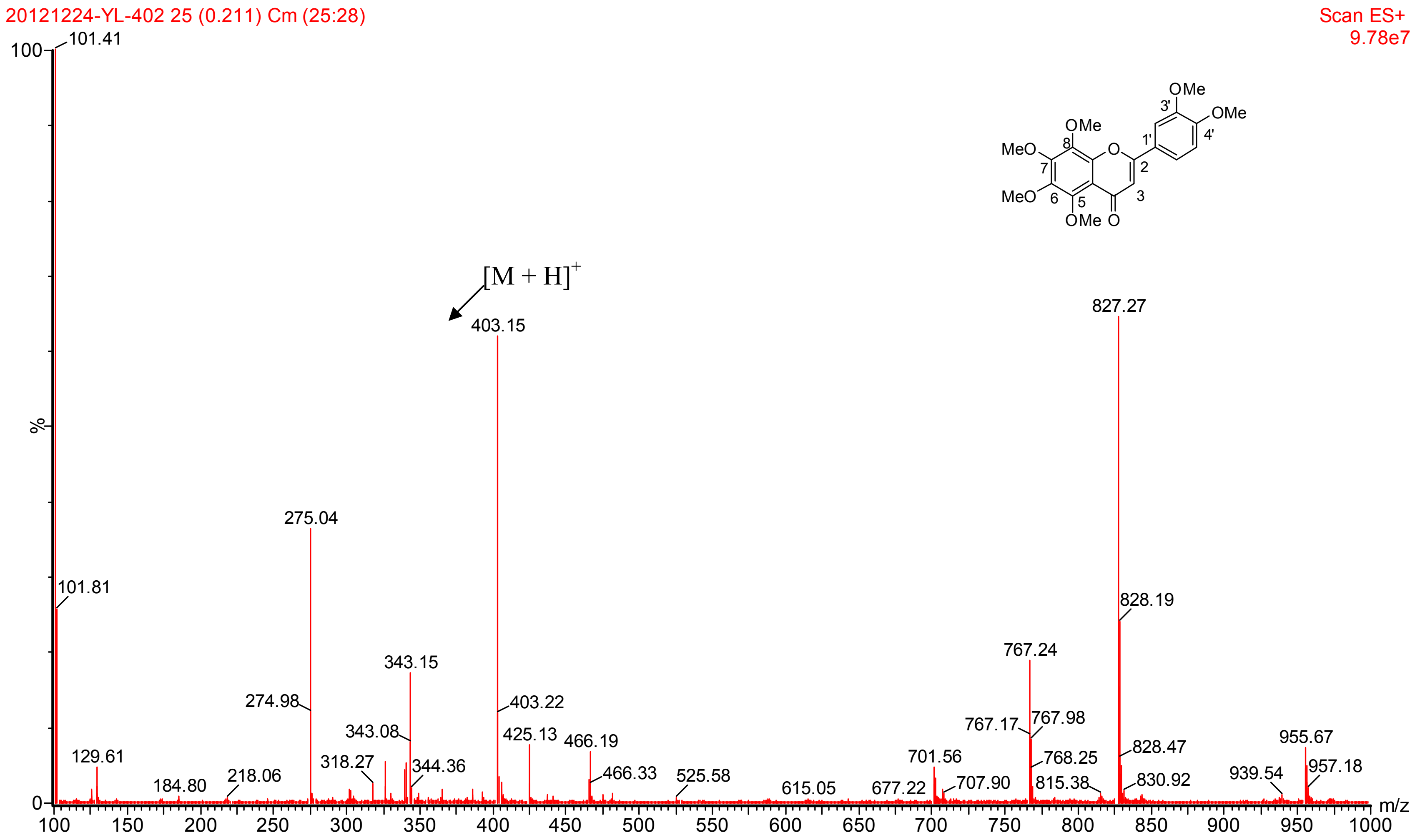

**Figure S36.** The $^{1}$H-NMR Spectrum of Compound **5** in $Me_2CO$–$d_6$.

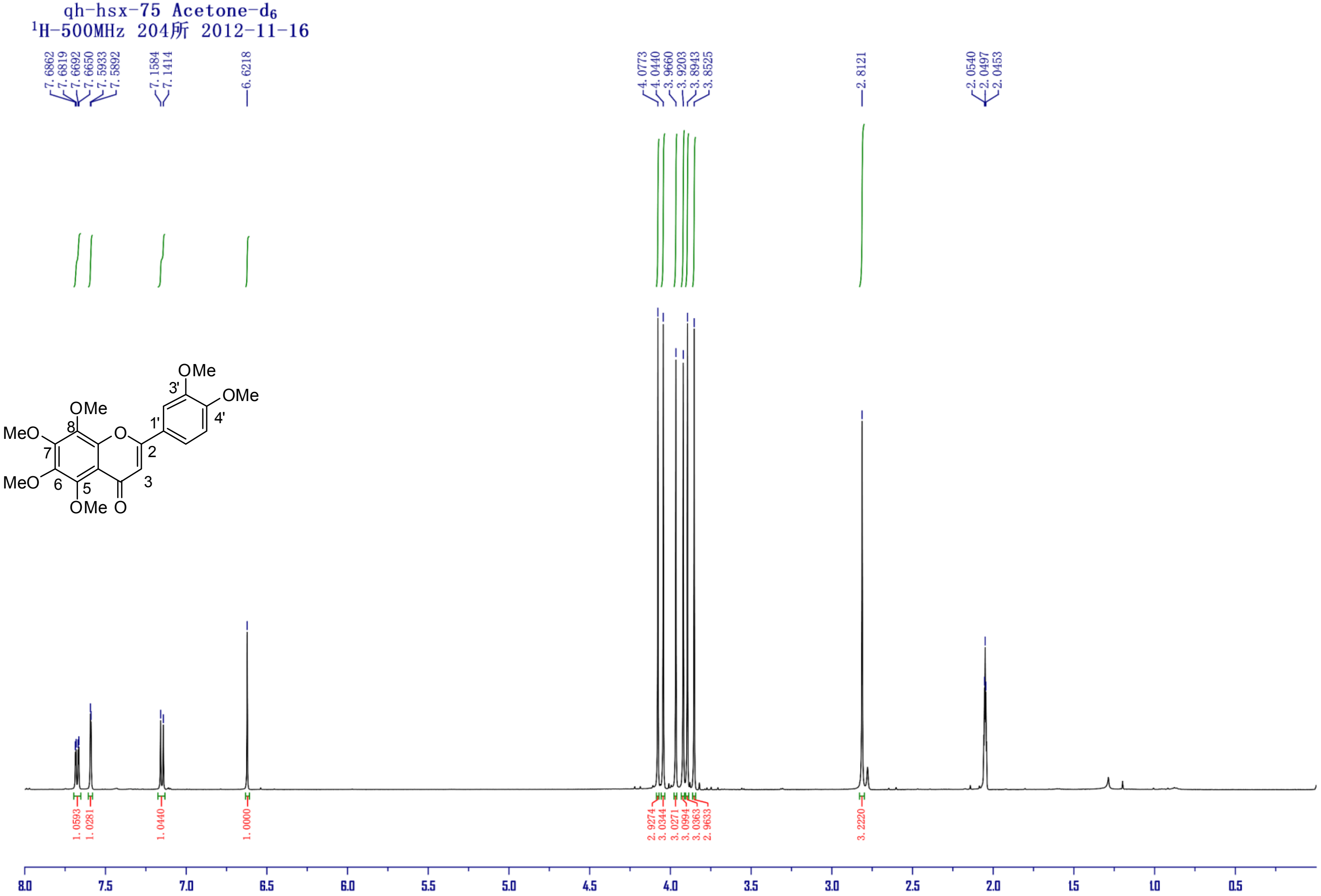

**Figure S37.** The $^{13}$C-NMR Spectrum of Compound **5** in $Me_2CO$–$d_6$.

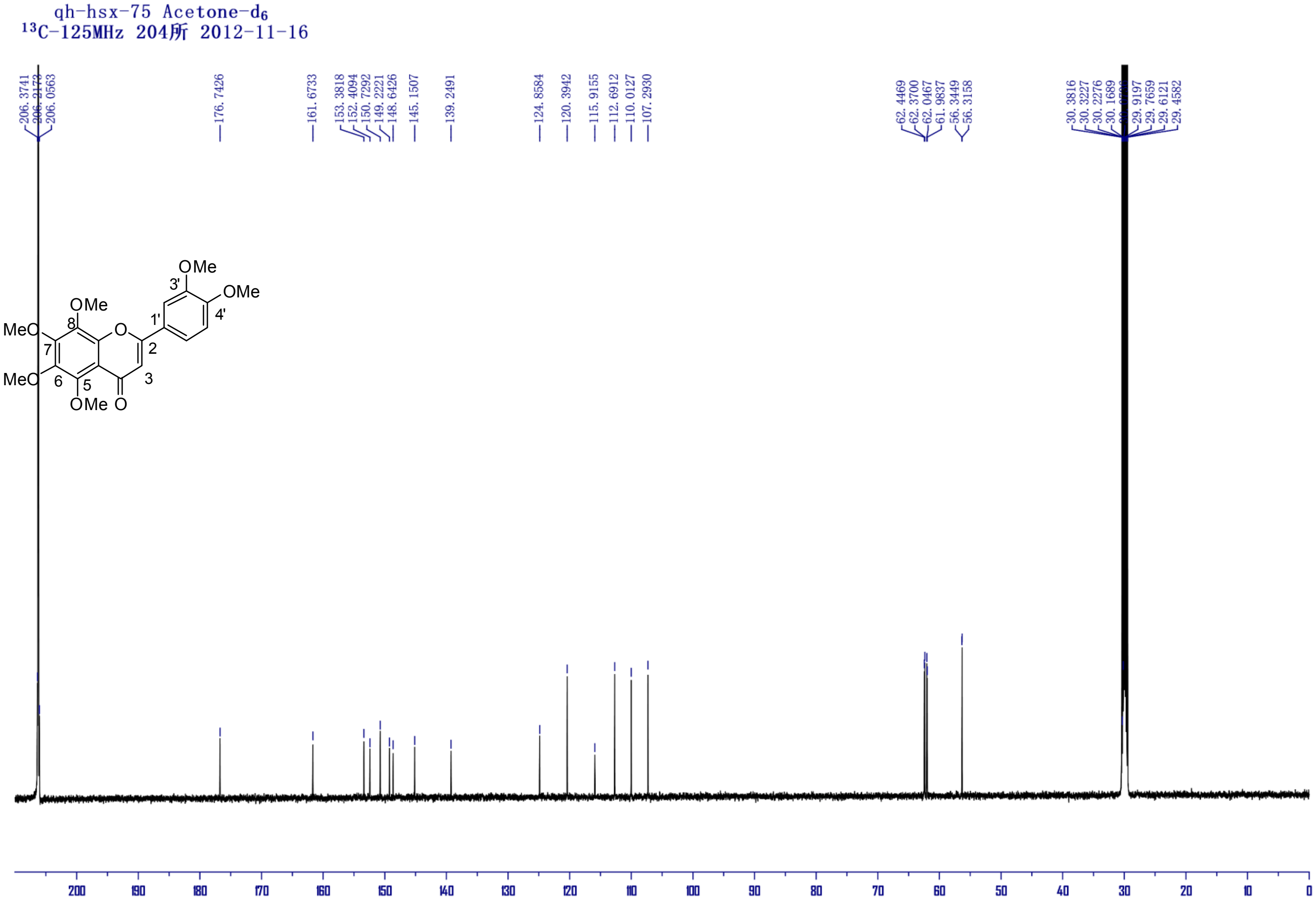

**Figure S38.** The DEPT Spectrum of Compound **5** in $Me_2CO{-}d_6$.

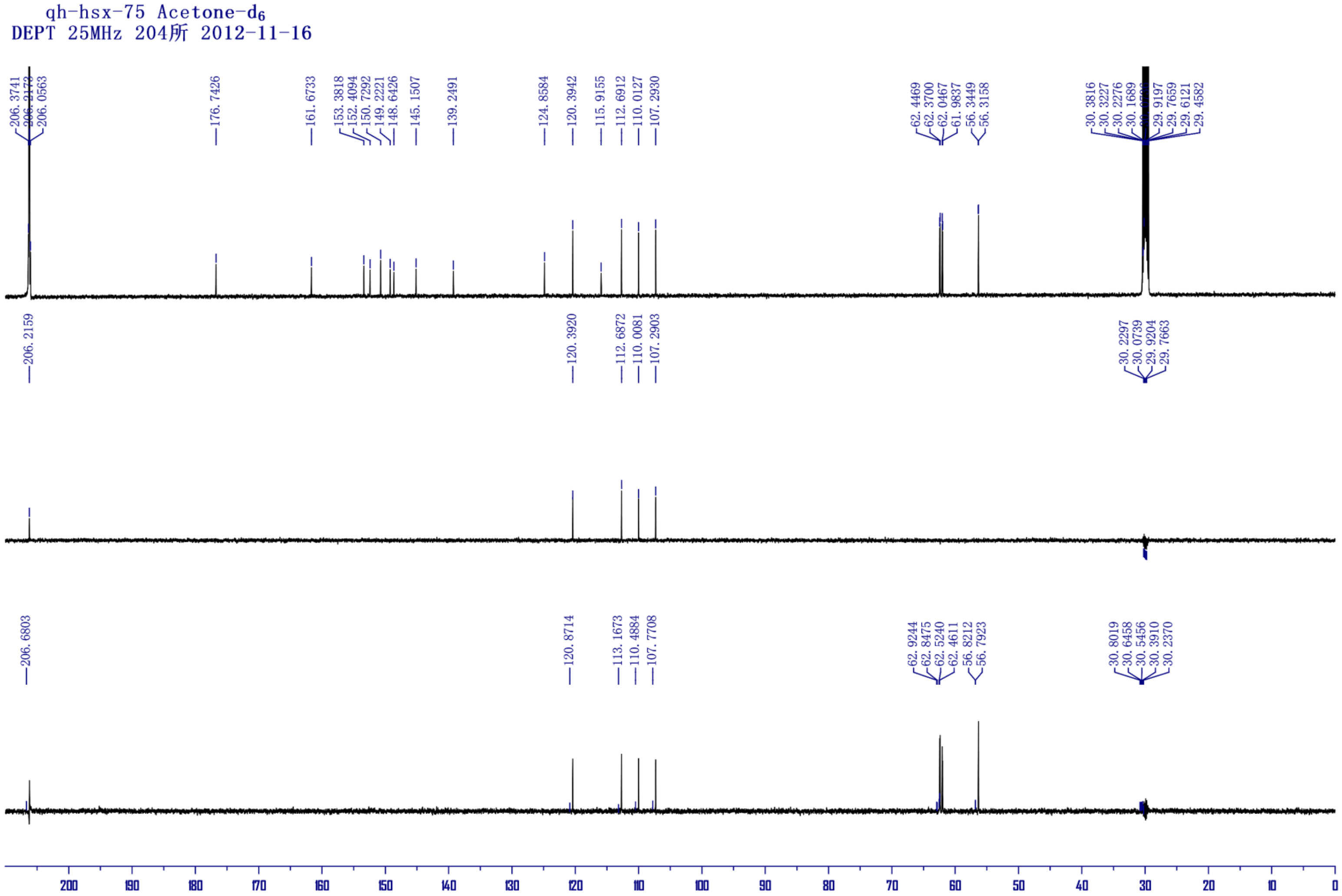

Supplement: Supplementary file 1 [file molecules-18-04766-s001.pdf]
